# Engineered Nanofiber-Hydrogel Systems for Colorimetric Lactate Sensing from Breath

**DOI:** 10.1021/acsami.5c15741

**Published:** 2025-11-06

**Authors:** Barbara V. Grotz, Klara Rogalla von Bieberstein, Nongnoot Wongkaew, Axel Duerkop, Margaret W. Frey, Antje J. Baeumner

**Affiliations:** a Institute of Analytical Chemistry, Chemo- and Biosensors, 9147 University of Regensburg, Universitaetsstrasse 31, Regensburg 93053, Germany; b Department of Human Centered Design, College of Human Ecology, 5922 Cornell University, Ithaca, New York 14853, United States

**Keywords:** point-of-care diagnostics, wearable system, breath analysis, nanofibers, noninvasive sampling, colorimetric lactate detection

## Abstract

Current methods for detecting chronic airway inflammation, such as asthma, rely on complex procedures and specialized clinicians. Taking advantage of inherent nanomaterial properties and their chemical design flexibility, nanofibers were designed and integrated with enzyme entrapping hydrogels. This composition offers noninvasive sample collection followed by simple colorimetric detection. Specifically, nanofibers were made from positively charged nylon-poly­(allylamine hydrochloride). They were optimized with respect to mat thickness, additive content, and lactate capture efficiency. The nanofibers could efficiently bind lactate through electrostatic interaction, correlating the resulting amount on the nanofiber mat to the concentration in breath aerosols. Detection was subsequently accomplished through a standard lactate oxidase, horseradish peroxidase assay with 3,3′,5,5′-tetramethylbenzidine colorimetric detection. The optimized nanofibers outperformed other polymeric nanofibers, face mask material, and filter paper regarding analyte capture and breathability due to the surface chemistry chosen and the high surface area afforded through the nanofiber mats. For lactate quantification directly on the mask, lactate oxidase was immobilized on the nanofiber mat via a hydrogel, ensuring long-term storage stability. Simple visual detection was achieved providing limits of detection of 5 μmol·L^–1^ (in solution) and 20 μmol·L^–1^ (hydrogel-based system) and a dynamic range that covers lactate concentrations found in breath, i.e., 5 to 150 μmol·L^–1^. This platform technology offers a promising solution for point-of-care diagnostics, contributing to remote healthcare, telemedicine, and simplified diagnostics in airway inflammation management.

## Graphical abstract

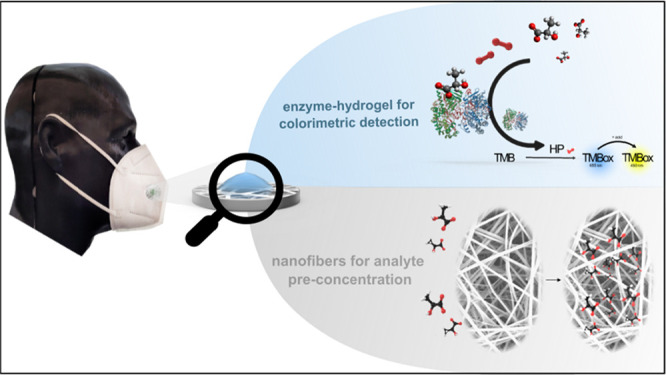

## Introduction

The COVID-19 pandemic highlighted the need for point-of-care (POC) detection in healthcare, particularly as rising costs and a shortage of specialized clinicians became increasingly evident. Additionally, the widespread adoption of face masks during the pandemic has initiated further mask developments, including masks designed for children.^1,2^ Using face masks for sampling of biomarkers represents the logical next step, enabling home use or application in a doctor’s waiting room. Such an approach opens new possibilities in healthcare, particularly in screening for diseases of the airways, where there is a direct correlation between lung function and analyte concentration in breath.^3–5^

Asthma, for example, remains one of the most common chronic diseases among children, with significant prevalence rates reported globally.^6^ According to the World Health Organization (WHO), underdiagnosis still represents a major challenge especially in developing countries, leading to undertreatment and aggravation of symptoms. Currently, asthma detection relies on expensive diagnostic equipment and specialized physicians. Rising healthcare costs and the limited availability of specialized clinicians highlight the urgent need for more accessible and cost-effective solutions for rapid asthma diagnosis. A POC test capable of identifying patients at risk of asthma would, hence, be highly beneficial.

Breath is a relevant analytical sample as a noninvasive matrix for disease detection, given its simple sample acquisition, suitability for all age groups, and unlimited availability, unlike blood, sweat, or urine. Lactate has emerged as a promising biomarker for the detection of airway inflammation from exhaled breath condensate, particularly in conditions such as asthma and chronic obstructive pulmonary disease (COPD). The metabolic changes in cells during inflammation result in increased lactate production, which can be measured to assess the severity of these conditions,^7–9^ making noninvasive real-time monitoring of local lung inflammation and disease progression through POC analysis possible. However, there are challenges to be addressed, such as the need for standardized measurement techniques and the potential variability in lactate levels due to factors unrelated to airway inflammation.^3^

Conventional POC detection methods for lactate, which operate in the millimolar range, are inadequate for the low concentration levels (low micromolar range) found in breath.^10^ Therefore, efficient sample preconcentration and extraction are essential.^3,8^ Nanofibers, with their high surface-to-volume ratio and lightweight nature present a promising solution for preconcentration of the analyte.^11^ Additionally, fabrication of nanofibers using electrospinning provides easy up-scaling possibilities.^1,12^ The efficiency of lactate preconcentration/filtration from exhaled breath can be enhanced using positively charged nanofibers making use of the electrostatic interactions between the additive in the fibers and the analyte.^13–17^ Electrospun nanofibers have not yet been applied to breath-based analyte preconcentration. However, nanomaterials are widely used in volatile organic compound (VOC) detection systems^18^ and face masks^19^ with growing interest in breath analysis due to their sensitivity and rapid response. Materials like WO_3_ (doped with Pt/Pd) and polyaniline blends detect biomarkers such as acetone, ammonia, and hydrogen sulfide, relevant to diabetes and halitosis.^18^ Despite increasing healthcare interest,^20^ clinical use remains limited due to challenges like poor reproducibility, slow recovery, lack of VOC standards, and confounding factors like smoking.^18^ Hence, most systems still rely on mechanical breath parameters rather than disease-specific VOC profiling or aerosol diagnostics.^21,22^

Therefore, we report here the development of the first point-of-care lactate diagnosis system to detect lactate directly from breath aerosol, combining nylon-poly­(allylamine hydrochloride)-nanofibers for analyte enrichment and preconcentration with a lactate assay for colorimetric determination of lactate based on the enzymatic reaction coupling lactate oxidase, horseradish peroxidase and the colorant 3,3′,5,5′-tetramethylbenzidine (Figure 1).^23,24^ Upon mounting such a nanofiber sheet into a face mask, a POC-device with the following performance characteristics is obtained: (i) convenient handling for the user through (ii) direct collection of the analyte from breath aerosol without the need for an exhaled breath condensate collection device and (iii) improved performance as well as comfort compared to other materials on the market. This could provide a rapid, cost-effective, and convenient solution for asthma detection in the future, enabling early intervention and improved patient outcomes.

**Figure 1:**
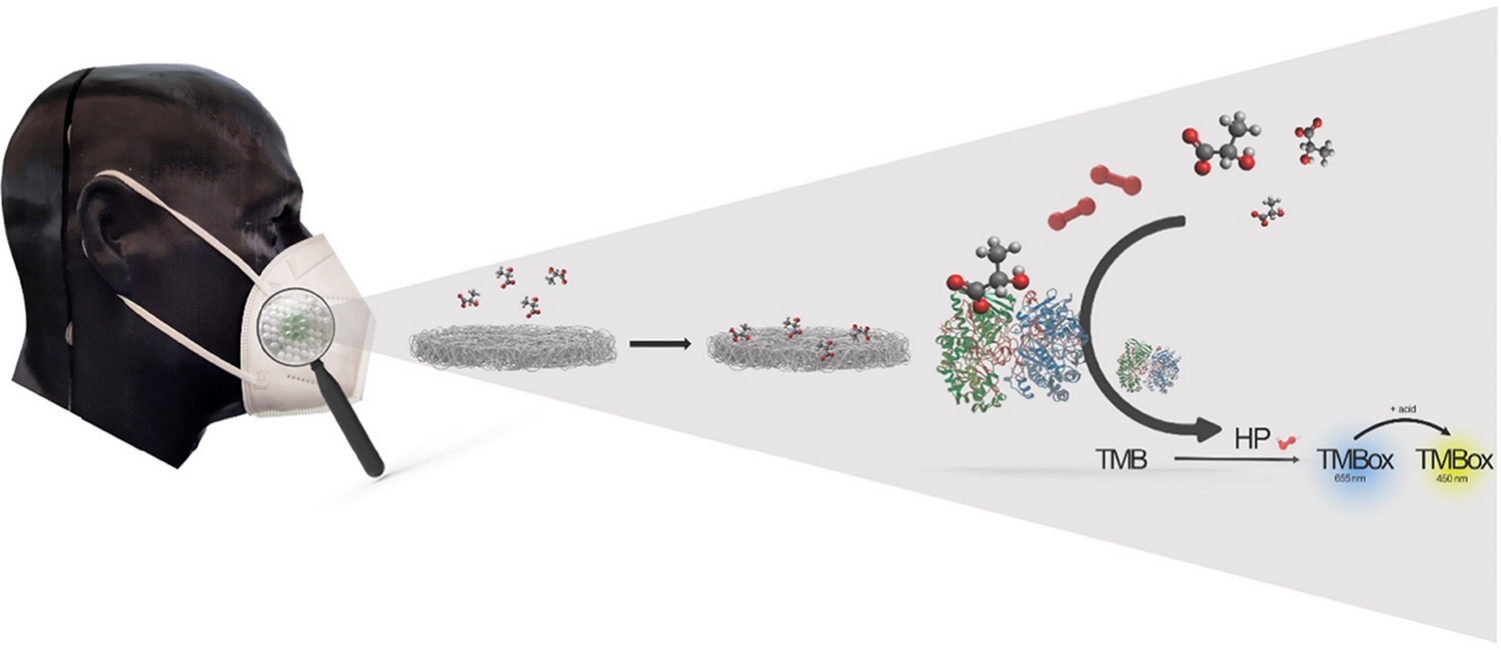
Schematic sketch of a nanofiber-hydrogel patch integrated in a face mask for lactate capture and detection via an enzyme-based colorimetric reaction.

## Materials and Methods

### HRP-Based Colorimetric Lactate Assay

A horseradish peroxidase (HRP, Sigma-Aldrich) assay using 3,3′,5,5′-tetramethylbenzidin (TMB, Sigma-Aldrich) was used for the colorimetric detection of lactate. Lactate oxidase (LOx, Hoelzl Diagnostics, and Sorachim) was incorporated into the system to allow enzymatic detection of lactate. Sodium lactate (Sigma-Aldrich) solutions of different concentrations were prepared and subsequently nebulized (Portable Mesh Nebulizer Air Q+ from Feellife Health Inc., photo in the SI), inducing the formation of lactate containing aerosol mimicking exhaled breath. Experiments were performed with lactate concentrations representative of those found in exhaled breath condensate (EBC): 0–500 μmol·L^–1^.^25^ A TMB solution of 1 mM was prepared from a 15 mM TMB stock solution in DMSO via dilution in DI water. Enzyme concentrations of 2 mU·μL^–1^ LOx and 0.2 mU·μL^–1^ HRP were obtained via two-step dilutions of the respective stock solutions: 10 U·μL^–1^ for LOx (via 2 U·μL^–1^) and 0.01 U·μL^–1^ for HRP (via 2 mU·μL^–1^). For LOx, the stock solution was stored at −20 °C, the HRP stock solution and the respective dilutions were stored at 4 °C. Enzyme stock solutions were prepared by dissolving the respective amount of enzyme in DI water.

### Nanofiber Fabrication

Nylon-6,6 (*M*_W_ ≥ 226.14 g/mol, Sigma-Aldrich) nanofibers with positively charged additives, polybrene (PB, Sigma-Aldrich) or poly­(allylamine hydrochloride) (PAH, M_W_ 50,000, Sigma-Aldrich), were produced by electrospinning. Therefore, 12 wt % nylon-6,6 and the respective amount of the additive were dissolved in formic acid through stirring for 12 h at room temperature (RT) until a homogeneous spinning solution was obtained. Different concentrations of PAH or PB (1.2, 2.3, 3.5, 4.6, 5.5 wt %, with respect to the total polymer solution) were investigated. The resulting nylon-6,6-PAH and nylon-6,6-PB solutions were used for electrospinning of nylon-PAH nanofibers or nylon-PB nanofibers, respectively. Each resulting solution was loaded to a 5 mL-glass-syringe with a metallic 20 G needle. During electrospinning a flow rate of 2 μL·min^–1^ was selected and maintained with a syringe pump. A voltage of +21 kV was applied through a high voltage supply (Linari Engineering srl, Pisa, Italy). Randomly oriented nanofibers were deposited on a sheet of grade 1 chromatography paper (Watman) fixed on a grounded rotary drum collector (Aluminum thin wall, diameter of 80 mm, and length of 120 mm, Starter Kit-Aligned 40 kV, Linari Engineering srl, Pisa, Italy) with a rotation speed of 150 rpm. The fiber collection distance between the collector and the end of the tip was fixed at 20 cm. A spinning time of 7 h was selected (5, 7, and 12 h were compared). The electrospinning process was carried out at RT and 35% relative humidity in a Plexiglas chamber (made in-house). Nylon-6,6 nanofibers without additive were prepared accordingly to serve as the reference material.

### Characterization of the Nanofibers and Reference Materials

The nanofibers were characterized by SEM images taken with a Zeiss/LEO 1530, Germany, 5 kV. Two 6 mm circles of nanofibers from different spots of the nanofiber mat were placed on an SEM holder. Then, the samples were sputtered with a gold–palladium mixture. SEM images from at least 8 different spots were taken. For the characterization of the nanofiber diameter, images with a magnification of 2500 k× (Image Pixel Size 10.78 nm) were used. Subsequently, nanofiber diameters were determined using ImageJ.

For contact angle measurements, a drop of 5 μL water was placed on the material (fiber mat, filter paper, medical mask, FFP2 mask, Owens & Minor HALYARD Global Products & Services) and an image was taken with a CCD camera on a Dataphysics contact angels system OCA 15EC and analyzed with ImageJ. The masks were cut into sections, allowing for a separate evaluation of each layer, as well as an assessment of the overall layering structure used in the masks.

### Enzyme Immobilization in Hydrogels

An enzymatic hydrogel matrix, based on medical grade polyurethanes (HydroMed D4, HydroMed D640, AdvanSource Biomaterials Mitsubishi Chemical America), was used to immobilize the enzymes HRP and LOx. Enzyme hydrogel solutions were obtained by adding the respective amount of the previously described HRP and LOx stock solutions to 1 g of previously prepared D4 or D640 hydrogel solutions (5 or 10 wt % in EtOH/H_2_O, 9:1, v/v). The solutions were used to produce polymer films on a Mylar support (Goodfellow, UK) with a knife-coater (Coesfeld Material Test, Dortmund, Germany) or directly drop-coated with direct displacement pipettes (Gilson, Microman, USA) to the wells of an MTP or to the freestanding nanofiber patches.

### Capturing Lactate Aerosol using Nanofibers

Freestanding nanofiber patches (nanofibers after removal of filter paper support) on PET support discs (laser-cut in specific breath-permeable design, Table S1, Figures S1–S3) were cut out of the nanofiber mats using a toggle-press. These nanofiber patches were integrated in a hand-held nebulizing device (Figure S5) or in the breathing pathway of a breathing apparatus (Figure S6) to interact with aerosolized lactate solutions. Captured lactate from aerosolized solutions was quantified using the enzymatic reaction of LOx and HRP, with absorbance measurements of oxidized TMB either via addition of the enzymes in solution (Figure S7) or with enzyme hydrogels drop-coated on the nanofiber patches.

### Measurement Parameters and Statistical Evaluation

Absorbance measurements at wavelengths of 655 and 450 nm were performed in transparent microtiter plates (96 wells, flat bottom, Greiner Bio-One International GmbH) using a plate reader Synergy Neo2 Hybrid Multi-Mode Reader (BioTek Instruments Inc. USA). Absorbance values were plotted in relative absorbance units. The obtained values were used as the ″capture efficiency” of lactate. For optimization experiments, 150 μmol·L^–1^ lactate was prepared in solution and nebulized. In comparison studies, the absorbance intensities were normalized to the initial measurement and the values are given in percentages. To determine the charge density on the nanofiber mats, freestanding NH_2_ groups were detected using a fluorescent CBQCA assay (CBQCA Plus Protein Quantitation Kit, Thermo Fisher Scientific), see Table 1 for parameters used. *n* = 3 or 4 was used in all experiments, if not stated otherwise. The mean value over *n* is plotted with the standard deviation obtained via eq 1.

$$s=\surd \left[\frac{\Sigma (x_{i}-x®{)}^{2}}{\left(n-1\right)}\right]$$ 1

where *s* is the sample standard deviation, Σ is the sum over all data points, *x_i_* is the individual data points, *x̅* is the sample mean, and *n* is the number of data points in the sample

**Table 1:** Parameters and Settings of Measurements Performed with the Plate Reader

parameter	HRP/TMB assay	CBQCA assay
measurement	absorbance end point	fluorescence end point
temperature	25 °C	25 °C
shake	567 cpm	360 cpm
	0:10 (MM:SS)	0:10 (MM:SS)
wavelengths	655 nm	ex: 465/20 nm
	450 nm	em: 550/20 nm
optics		top
gain		100

## Results and Discussion

Nanofiber mats were opimized for the capture of lactate directly from aerolyzed lactate solutions simulating breathing actions. Furthermore, two assay strategies were developed: focusing solely on sample preconcentration or integrating preconcentration with direct point-of-care readout, respectively. Hence, lactate-quantifying enzymes were added to the nanofibers in solution or directly immobilized in a hydrogel on the nanofiber mat. For the initial development of the nanofiber-based mat, all experiments were carried out using enzymes in solution. The overall concept is illustrated in Figure 1.

### Material Selection for Nanofibers and Proof of Concept

Nylon was selected as the base polymer for the fabrication of nanofiber mats due to its hydrophilic properties, biocompatibility,^26^ mechanical stability,^27^ and resistance to humidity,^28–30^ making it a good fit for the incorporation into face masks. To ensure a high lactate capture efficiency, nylon was doped with positively charged polymers either poly­(allylamine hydrochloride) (PAH) or Polybrene (PB), assigned as nylon-PAH or nylon-PB nanofibers, respectively (Table S1). The initial electrospinning parameters were adjusted for each additive separately, focusing on a high surface charge density and fiber stability. SEM imaging revealed that fibers with PB were broader (847 ± 382 nm) and flatter, whereas PAH-incorporated fibers exhibited more uniform morphologies (580 ± 240 nm) (Figure 2a, Figure S8). However, in both cases, nanonets were observed, which is an inherent characteristic of electrospun nylon nanofibers. The nanonets have exibited their superiority in nucleic acid extraction as demonstrated by our previous study.^31^

**Figure 2:**
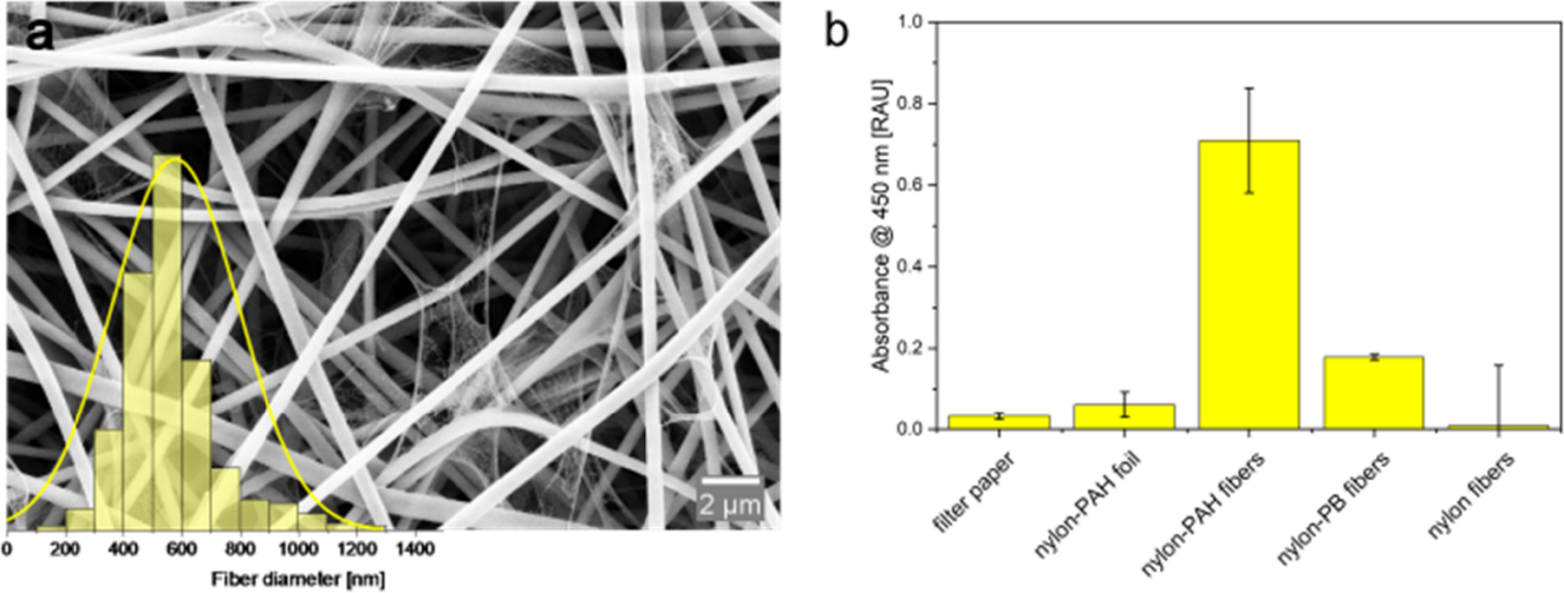
(a) Size distribution and SEM image for a magnification of 10.00 k× for nylon-PAH nanofibers obtained by electrospinning with a mean diameter of 578 ± 216 nm. *n* > 200. (b) Adsorption efficiency of lactate on various capture materials after 2 min incubation with 150 μmol·L^–1^ nebulized lactate, measured via LOx+HRP/TMB assay (@450 nm). Nylon-PAH nanofibers were compared to filter paper, nylon-PAH polymer foils, nylon-PB nanofibers, and pure nylon nanofibers. *n* = 4.

Captured lactate from aerosolized solutions was quantified using the well-known enzymatic reaction of LOx and HRP, with absorbance measurements of oxidized TMB. For more details on the optimization of the assay, please see the respective description in the SI. The assay showed that nylon-PAH nanofibers outperformed nylon-PB in lactate capture, likely due to their highly accessible positive charge (Figure 2b). Comparing the nylon-PAH nanofibers to the same composite polymer cast as a film, the nanofiber mat outperformed the film in lactate capture by a factor of 12, highlighting the advantage of the high surface-to-volume ratio and their breathability (Figure 2b). While hydrophilicity supports analyte interaction by improving wetting and diffusion, it is not the sole determining factor. For instance, filter paper, despite being more hydrophilic than the nanofiber mats (Figure S9), showed significantly lower capture efficiency, likely due to its unspecific absorption of the sample matrix and lack of electrostatic binding sites required for targeted analyte retention. However, no BET analysis was performed since one squaremeter of nanofiber mats, equaling 270 h of electrospinning, would have needed to be produced for a single measurement in the setup available to us. Furthermore, this nonspecific gas adsorption measurement would not necessarily correlate to the charge-based capture targeted here.

Nylon-PAH nanofibers uniquely combine hydrophilicity, high surface area, and positively charged PAH residues, enabling specific electrostatic interactions with lactate, which carries a negative charge at physiological pH. This is further supported by comparisons to nylon nanofibers without PAH, which despite similar morphology and surface area exhibited a much lower capture efficiency (Figure 2b). Similarly, nylon-PAH films, although containing the same charged material, show reduced capture efficiency due to their limited surface area. In summary, the superior performance of nylon-PAH nanofibers over films and filter paper arises from the interplay between morphology (surface area), surface charge density, and hydrophilicity. Each factor contributes to capture efficiency, but only in combination do they result in the high performance observed. Hence, nylon-PAH nanofibers were chosen for further development.

Electrospinning conditions were systematically optimized to balance fiber mat thickness and capture efficiency, with spinning times of 5, 7, and 12 h and additive (PAH) concentrations of 0.3, 1.4, 2.4, and 3.8 wt %. Mat thickness was primarily controlled via spinning time, which influences performance through two key mechanisms: (i) improved mechanical stability and handling due to increased robustness of thicker mats and (ii) enhanced surface area for analyte interaction, particularly relevant for small molecules like lactate that can penetrate deeper into the fiber network. However, beyond a spinning time of 7 h, no further improvement in mat performance was observed, likely due to the insulating effect of accumulated fibers, reducing the efficiency of the grounded collector.

Extended spinning times (e.g., 7 and 12 h) produced mats with good mechanical stability (data not shown). Specifically, mats spun for 7 h showed no change in fiber morphology after exposure to aerosol and mechanical bending in the breathing pathway, as supported by SEM imaging (Figure S10). Regarding additive content, lower PAH concentrations (e.g., 1.4 or 2.4 wt %) reduced the formation of freestanding fibers during production, indicating improved spinnability. The influence of PAH on the fiber morphology and capture efficiency is attributed to its role in modulating the charge density of the polymer solution. While increasing PAH concentration generally enhances surface charge availability, thereby improving analyte capture, this effect reaches a plateau once surface charge saturation is reached, as indicated by CBQCA assay results detecting surface amino residues (Figure S11) and absorbance signals in LOx + HRP/TMB assays (Figure S12d).

Moreover, excessive additive concentrations (e.g., 3.8 wt %) can increase solution conductivity beyond optimal levels, negatively impacting fiber morphology by increasing fiber diameter and introducing structural artifacts (Figure S6). Based on these observations, a PAH concentration of 2.4 wt % and spinning time of 7 h were selected as optimal, providing an optimized balance between spinnability, mechanical stability, and capture efficiency of the nanofiber mats.

Reproducibility tests confirmed consistent production of nanofiber mats, even under variable laboratory conditions (e.g., different humidity and temperature, Figure 3a) even including serendipity of production over the course of several months, as nanofiber mats exhibited comparable absorbance signals and coefficients of variations (CVs) of below 10% (Figure 3b). Stability tests revealed no observable degradation or functionality loss over 8 months, as older fibers performed comparably to newly spun fibers (data not shown), and no morphology changes were observed in SEM pictures (Figure S10). These results confirm the robustness, functional stability, and reproducibility of the developed nylon-PAH nanofibers, indicating sufficient viability for industrial applications.^31^ Stability testing of lactate on the nanofiber mats (3 min, 5 min, 10 min, 30 min, 1 h, 3 h, 24 h, and 1 week) confirmed the system’s applicability for POC applications. Specifically, nylon-PAH nanofibers were found to effectively capture and stabilize lactate via electrostatic interactions, maintaining consistent signals for at least 1 week (Figure S13). Pure nylon nanofiber mats had a significantly lower capture efficiency than nylon-PAH fibers (Figure 2b), highlighting the necessity of positive charges for lactate capture. Previous studies^31^ showed that nylon-PAH nanofibers are positively charged between pH 4.5 and 10, enabling strong interactions with negatively charged species. PAH’s p*K*_a_ (8–9.5) varies with ionic strength, molecular weight, and solution conditions, but in physiological breath (pH 6–8), PAH remains mostly positively charged, while lactic acid (p*K*_a_ 3.76, NIST) exists primarily as anionic C_3_H_5_O_3_^–^.^32^

**Figure 3:**
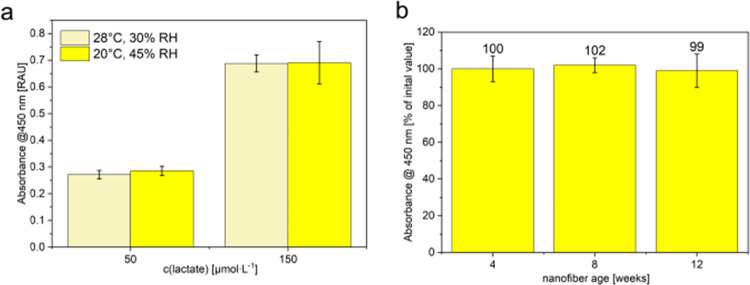
(a) Investigation of the reproducibility of nanofiber production for different relative humidity values (30% RH and 45% RH) and different temperatures (20 and 28 °C). All other electrospinning parameters were kept constant. (b) Stability of nylon-PAH-nanofibers over the course of 3 months (4, 8, and 12 weeks) given in percentual values of the initial absorbance intensity obtained directly after nanofiber fabrication. The same nanofiber mat was used for all of the experiments in this study. Absorbance measurements @450 nm after 3 min incubation of freestanding nylon-PAH nanofibers with 150 μmol·L^–1^ nebulized lactate and lactate detection in LOx + HRP/TMB assays. *n* = 3.

### Performance of Nanofibers for Lactate Capture and Breathability

The optimized nylon-PAH nanofibers with 2.4 wt % PAH were compared to conventional materials such as filter paper, FFP2 masks, and medical masks, assessing the overall combination of lactate capture and breathability. Lactate capture efficiency was assessed by using the LOx + HRP/TMB assay (Figure 4a) and was the highest for the nanofibers. Compared to this benchmark, filter paper achieved only 39%, FFP2 masks 25%, and medical masks just 8% of the nanofiber capture efficiency. Furthermore, breath droplet permeability was measured via a particle counter, and breathability was evaluated via the backpressure measured using a hand-held manometer breathability (Figure 4b, Figure S6). The nanofibers also demonstrated the lowest breathing resistance: breathing through FFP2 masks was 1.4 times harder, through medical masks 1.8 times, and through filter paper 3.2 times harder than through the nanofiber material, highlighting its dual advantage of high capture efficiency and superior breathability. Contact angle measurements (Figure S9b) showed similarities of nanofiber hydrophilicity to that of filter paper. The droplet capture efficiency of the nanofibers was in a similar range to the commerical mask material (Figure S9a). As a result, the nanofiber mats outperformed all other materials with respect to both lactate capture and breathability. As polymers used in face mask production are mostly uncharged and hydrophobic,^33^ the results are not too surprising but also demonstrate the significant advances that can be made in the use of face masks for analyte capture and detection through capture material optimization.

**Figure 4:**
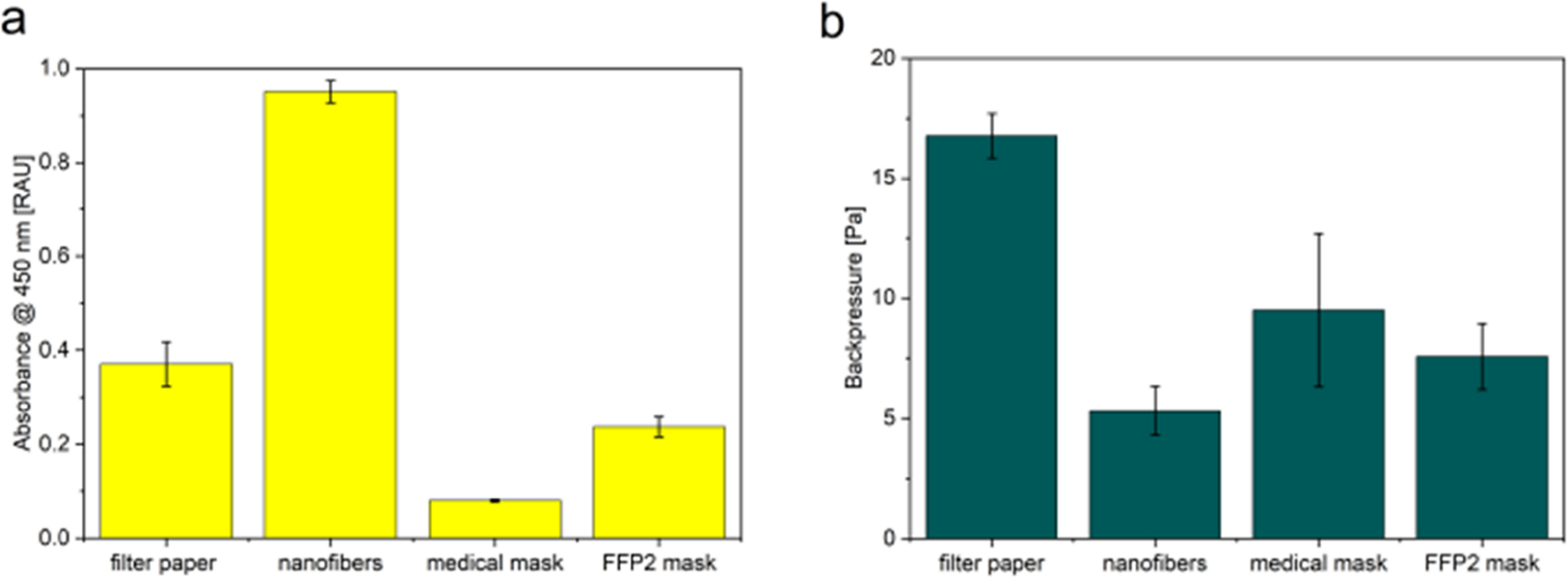
Comparison of developed nanofiber material to commercially available mask materials and filter paper with respect to (a) lactate capture efficiency and (b) breathability of the materials. Experiments were conducted in the simulated breath apparatus with 100 μmol·L^–1^ lactate and a sampling time of 5 min with 25 breaths/min and a 50/50 inhalation-to-exhalation ratio. *n* = 4.

### Optimization of Sampling Conditions for Lactate Capture

Tests were conducted under simulated breathing conditions to assess the assay’s sensitivity to variations in breath rate (15, 25, 35 breaths/min) and inhalation-to-exhalation ratios (25/75, 40/60, 50/50). Preliminary testing was performed with a modified face mask to assess lactate capture for different positions in the breathing pathway. Variations in breath rates did not significantly impact the signal intensity (Figure S14b). This is beneficial, as asthmatic patients usually breath with higher frequencies (e.g., healthy population: between 15 and 25 breaths/min vs asthmatic patients with 30 or more breaths/min).^34,35^ Exhalation-dominant breathing patterns, however, increased sample contact with the nanofibers and enhanced signal output underlining the need for normalized breathing instructions for assay application and a development of an internal control in the future (Figure S14a).^5^ Sampling times were optimized, indicating that a 5 min exposure is a good compromise between sampling duration and absorbance yield (Figure S14c). While effective aerosol sampling with nanofibers was achieved in laboratory settings, this duration will need adjustment for actual breath sampling in future studies as analyte levels may vary during breathing cycles compared to the constant exhalation provided by the nebulizer device.^5^ A 5 min sampling time already allowed the differentiation of breath-related concentrations of 50 μmol·L^–1^, 100 μmol·L^–1^ and 200 μmol·L^–1^ of lactate, respectively (Figure S14d,e).

### Development of a Point-of-Care Ready Immobilized-Enzyme Assay

To create an easy-to-use assay setup, the enzymes were incorporated into the nanofiber mats using hydrogels. Here, only TMB and sulfuric assay solutions are added after lactate capture, simplifying the overall assay significantly. Various polyurethane-based hydrogels of the HydroMed series^36^ were selected as the immobilization matrix (not all data shown) with D4 and D640 providing the most consistent results. Parameters such as polymer matrix (D4 vs D640), polymer concentration (10 wt % vs 5 wt %), enzyme concentration (0.005 wt %, 0.01 wt %, 0.05 wt %, 0.1 wt %, 0.5 wt %), enzyme ratio, drying time (1 h, 3 h in polymer foils/10 min, 30 min for hydrogel drops), and thickness (30 μm, 50 and 100 μm) were optimized in initial optimization experiments performed using knife-coated polymer films. An enzyme concentration of 0.05 and 0.1 wt % and a wet-layer thickness of 50 μm were identified as optimal (Figure S15). Overall, the total enzyme amount emerged as a key parameter, whereas the layer thickness had only a minor impact on the results suggesting that diffusion limitations were negligible in experiments with the analyte in solution (Figure S15). Optimization experiments of TMB stock solutions and dilutions across different solvents and dilution matrices further increased signal intensities, where the solvent DMSO mixed with DI water or citrate buffer were identified as most suitable (Figure S15b and S16). Furthermore, drop-coating experiments comparing single and multiple drop designs, volume (10, 25, 50 μL), different hydrogel base polymers, nanofiber coverage, diffusion limitations, and reproducibility studies were performed. The experiments highlighted that a single larger drop of hydrogel on freestanding nanofibers achieved superior results to multiple smaller drops (Figure S17). Also, the presence of bare, freestanding nanofibers around the hydrogel spot was found crucial to allow efficient interactions between the analyte in the breath aerosol and the nanofibers, which enhance lactate capture efficiency. However, achieving consistent drop coating proved challenging (Figure S18), reinforcing the preference for a one-drop approach over multiple drops. Notably, more viscous hydrogel solutions (15 wt % vs 10 wt %) resulted in smaller standard deviations, highlighting the impact of drop-coating reproducibility and hydrogel drop stability, as indicated by their consistent size.

Finally, D640-hydrogel (10 wt %) exhibited increased sensitivities compared to D4 (5 or 10 wt %) with limit of detections (LODs) of 5 and 15 μmol·L^–1^, respectively (Figure S9a). It also showed enhanced enzyme storage stability (Figure S19), likely due to its increased water content, which increases pore sizes, enhancing enzyme mobility. While this increased rotational freedom has a positive impact on enzyme functionality and stability, no to little impact of enzyme leakage on long-term performance was observed (Figures S19–S21). HRP remained stable and functional for at least 16 weeks, exceeding the commonly reported 30–40 days in the literature already.^37–39^ In the case of LOx, the well-known low long-term stability of 14 to 49 days^39–41^ was also observed here as its stability declined after 4 weeks (Figure S20b). The addition of human serum albumin (HSA) as a stabilizing agent,^42,43^ had no significant impact on LODs or short-term enzyme stability (Figure 6b), though long-term effects need to be studied in the future.

### Comparison of the Two Assay Strategies

After the overall development of the nanofiber mat, the two possible assay strategies for lactate quantification (solution-based vs immobilized enzymes shown in Figure 5) were compared. As in the final setup, lactate containing breath aerosol were collected in the face mask, and all conditions were studied here by incubating nanofiber patches with aerosolized lactate.

**Figure 5:**
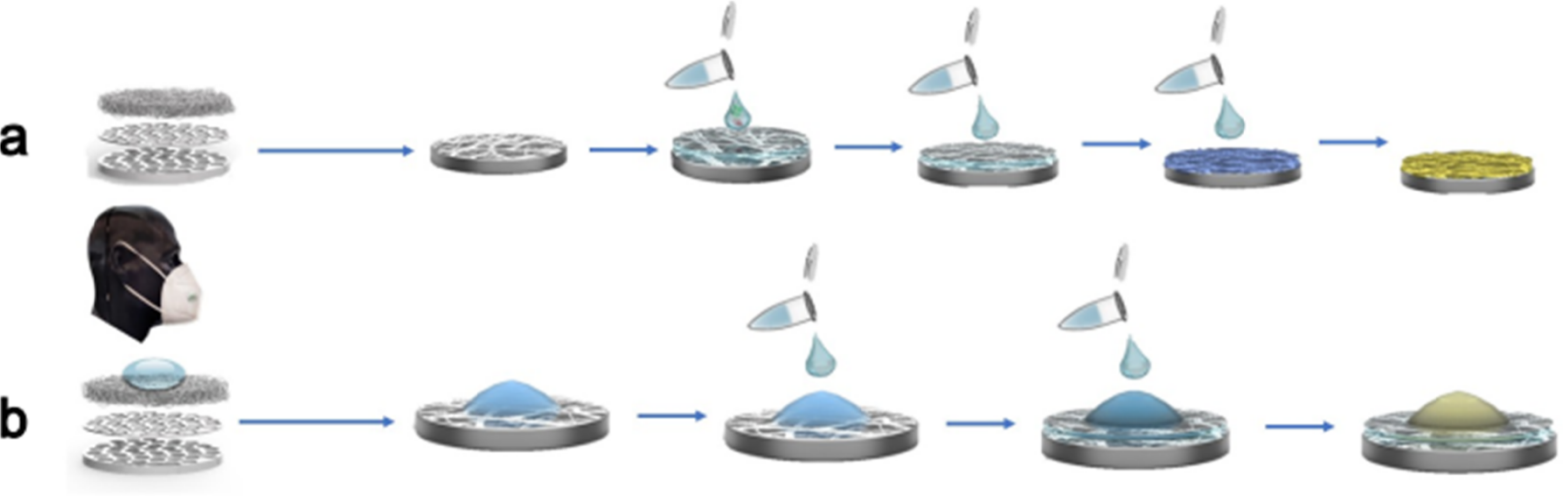
Schematic overview over the two pathways for the colorimetric assay of patients after sample collection on nylon-PAH nanofibers integrated into a face mask: (a) assay with enzyme solutions and (b) assay with enzyme hydrogel.

The optimized solution-based lactate assay in Figure 5a provided an LOD of as low as 5 μmol·L^–1^ with a dynamic range of 20 to 200 μmol·L^–1^ (Figure 6c). With lactate concentrations in breath between 5 and 150 μmol·L^–1^,^44^ this sensor system would be addressing the clinically relevant range.^9^ Furthermore, the assay demonstrated stable performance in the presence of potential interferents and matrix components—including lactic acid, glucose, ethanol, hydrogen peroxide, ammonia, acetone, BSA, and buffer salts (Cl^–^, Ca^2^^+^, K^+^, Na^+^, NO_3_^–^) across a pH range of 6 to 8—with minimal signal deviation and no significant impact on nanofiber integrity (Figure S22, Table S3). We employed this mixture of representative breath components, which can be termed ″artificial breath aerosol”, to simulate the matrix. This approach allowed us to systematically evaluate assay robustness under conditions mimicking breath composition. Based on these promising results, the assay can be validated with real patient samples in the future. In the case of the hydrogel-based assay according to Figure 5b, an LOD of 77 μmol·L^–1^ (Figure 6a) was obtained which could be improved 4-fold to 19 μmol·L^–1^ by simply increasing the LOx concentration by a factor of 4 (Figure 6b). It is assumed that the increased diffusion limitations for lactate and hydrogen peroxide in the hydrogel and the electrostatic immobilization of lactate on the nanofibers restrict their effective interaction with enzymes inside the hydrogel and hence lead to this increased LOD (Figure 6a,b compared to Figure 6c,d). While glucose detection relies solely on enzyme–analyte interactions, the lactate system benefits from electrostatic interactions that efficiently capture and accumulate analyte from breath aerosol over time, thereby up-concentrating the target and significantly enhancing system sensitivity (10 to 30-fold increase compared to glucose, Figure S23)

**Figure 6:**
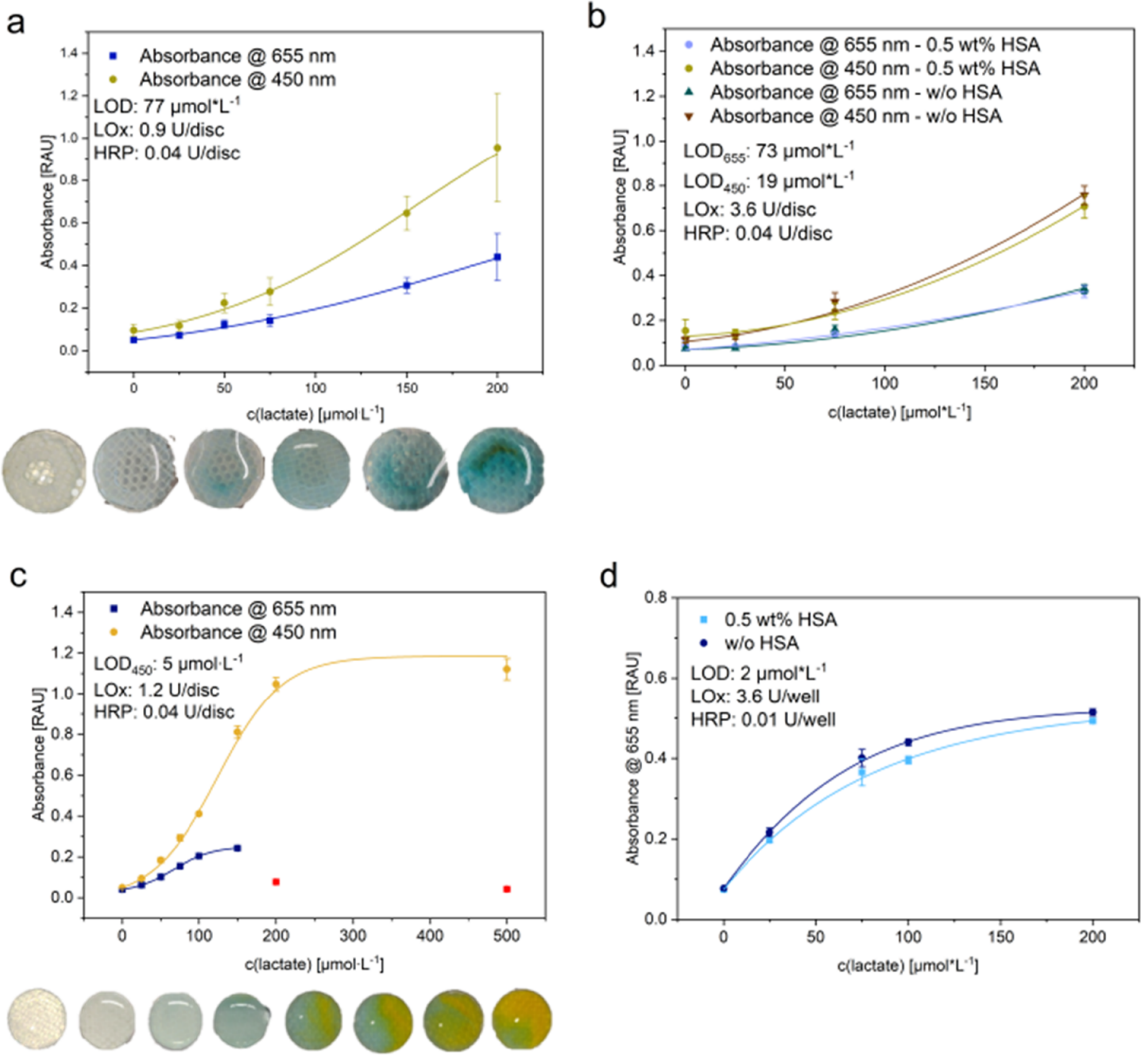
Influence on the LODs/absorbance yield of lactate-LOx+HRP/TMB assays of (a) nylon-PAH-nanofiber-LOx/HRP-hydrogel combination, (b) HSA addition and increased LOx concentration in nanofiber-LOx/HRP-hydrogel combinations, (c) nylon-PAH-nanofibers with enzymes in solution, and (d) LOx/HRP-hydrogel with lactate in solution. Pictures of nanofiber patches in the respective assays, before acid addition (a, c). The red squares in panel (c) indicate values marked as outliers, as an orange coloration was observed instead of the blue product for high analyte concentrations.

### Comparison to Existing Systems

Lactate sensing is well-established across biological matrices, such as blood, plasma, and sweat, with various commercial systems offering rapid detection. Blood-based devices like the **Lactate Plus** (**nova biomedical**), **Lactate Scout Sport** (**EFK Diagnostics**), and **Lacto Spark** (**B-Arm**) deliver results within 5–13 s using minimal sample volumes (0.2–0.7 μL) and cover detection ranges suitable for clinical and athletic use (typically 0.5–25 mmol·L^–1^). These systems employ electrochemical biosensors and are widely used in POC and sports monitoring. In research settings, advanced electrochemical techniques have achieved blood lactate detection limits as low as 0.7 ^45^ or 7.9 μmol·L^–1^ ^46^ (Table 2).

**Table 2:** Overview of Existing Publications in the Field of Lactate Detection^a^

matrix	mode of detection	response time	LOD [μmol·L^–1^]	publication date	ref
blood	amperometric	90 s	0.7	2008	^45^
blood plasma	amperometric	5 s	7.9	2024	^46^
blood, EBC	contactless conductivity detection	2 min collection of EBC: 1 min	0.392	2016	^49^
blood	electrochemical	13 s			Nova Biomedical
blood	enzyme-based, amperometric	10 s	range: 500–25,000		EKF Diagnostics
blood	amperometric	5 s	range: 200–25,000		B-Arm
sweat	enzyme-based electrochemical	continuous	range: 1000–50,000		IDRO
sweat	aptamer-based, electrochemical	continuous			Sensate Biosystems
sweat	colorimetric	30 s	1000	2022	^47^
(artificial) sweat	colorimetric	4 min	69	2021	^58^
saliva, serum	colorimetric/fluorometric	10 min	100	2020	^59^
sweat	colorimetric		60	2021	^60^
(artificial) blood, sweat, milk	colorimetric	10–15 min	0.17	2024	^53^
artificial saliva	colorimetric	<30 min	5700	2024	^61^
breath aerosol	colorimetric	5 + 10 min sampling + detection	in solution: 5 in hydrogel: 20		this work

a Different matrices, modes of detection, response times, and limits of detection are compared.

Sweat-based wearables such as the **IDRO Patch** and **Sensate Biosensor** enable continuous, noninvasive monitoring, making them more suitable for long-term tracking than instant diagnostics. Research toward colorimetric sweat-lactate sensors strategies increases, already providing approaches for wearable, real-time monitoring, albeit with higher detection limits of 1 ^47^ and 6.4 mmol·L^–1^.^48^

In contrast, breath-based lactate sensing remains under development with no commercial test being available to date. Techniques like contactless conductivity detection^49,50^ and LC-MS/MS^51^ have achieved detection limits down to 0.1 μmol·L^–1^ but require complex instrumentation and long processing times, limiting their practicality for POC use. Innovations such as by Zhang et al.^52^ have shown promise with nanomolar-range detection limits and simpler protocols for EBC analysis. Despite these advancements, challenges, such as improving portability and reducing assay times persist. Strategies like preconcentration^8^ enhance sensitivity but increase system complexity, while multimatrix sensors^53^ require further optimization for breath-specific applications and raise environmental concerns due to copper reagents.

The system developed in this work addresses key challenges in breath analysis by combining direct analyte collection, effective preconcentration, and a straightforward colorimetric readout. The integration of nanofiber materials enables sampling directly in the breathing pathway, eliminating the need for exhaled breath condensation, pipetting steps, or specialized instrumentation. Detection limits as low as 20 μmol·L^–1^ were achieved using the enzyme hydrogel-based assay, with only 5 min of sampling and a 10–15 min incubation. The simplicity of the procedure allows operation by untrained personnel, making it particularly suitable for POC applications. Additionally, collected samples can be stored for up to 1 week, allowing face masks with nanofiber patches to be gathered and batch-analyzed in clinical settings, enhancing diagnostic practicality.

Compared to existing methods, this approach avoids extensive equipment,^51^ intricate fabrication processes,^48,53^ and laborious sample preparation steps,^10,50,54^ while maintaining a low estimated material and production cost of approximately $0.30 per assay (Table S4). Although electrochemical systems achieve lower detection limits,^10,45^ they typically involve higher costs and more complex readout requirements. In contrast, the presented system offers a cost-effective and scalable alternative with clear advantages, in terms of accessibility and ease of use. Importantly, future improvements in the drop-coating step, such as the implementation of automated nanospotting deposition techniques, are expected to further enhance sensitivity and reproducibility. Another way to further enhance sensitivity could be the incorporation of pyruvate oxidase to boost signal intensity.^53^

This could further improve the assay performance while maintaining its inherent advantages. Avoiding environmentally hazardous reagents and using scalable nanofiber and hydrogel technologies^55–57^ further positions this system for industrial production and regulatory approval in medical applications. Overall, this work presents a breath sensing platform that balances sensitivity, simplicity, and cost and offers strong potential for real-world POC diagnostics.

## Conclusions

In this study, we developed novel nanofibers tailored for efficient breath lactate sensing in face masks, which demonstrate superior performance in analyte capture and sensing compared to other tested materials. This remarkable performance is attributed to the high surface-to-volume ratio of the nanofibers and their porous structure with nanonets, which enables enhanced interaction with analyte molecules for efficient capture. In addition, the nanofibers showed excellent stability (>3 months) and reproducibility even though fabrication was manually done on a small lab scale under ambient conditions. Two assay strategies were investigated and optimized in which either the face mask would merely serve for lactate collection or also provide embedded signaling capabilities. In the future, the system can be adapted for the detection of other charged analytes in breath or alternative matrices, such as sweat or saliva. While further research is needed to establish lactate as a reliable breath biomarker for disease detection, this platform represents a significant step toward noninvasive POC-monitoring from breath aerosol. If required, the system’s sensitivity could be improved toward lower detection limits potentially via pyruvate oxidase integration for a dual-enzyme TMB/HRP approach. Additionally, a smartphone-based readout would enhance standard­ization and reduce human error. These advancements will enable more precise, user-friendly, and scalable wearable diagnostics, accelerating the adoption of noninvasive biosensing for personalized health monitoring and clinical applications.

## Supplementary Material



## Notes

Detailed information about the chemical structure of the investigated polymers, the production of the sensor discs with PET support, a detailed description of the lactate assay, the devices and measurement setups used, SEM pictures and absorbance/lactate capture measurements that show the impact of spinning parameters like additive content, spinning time, storage and analyte capture on nylon-based nanofibers, information on the charges available on the nanofiber surface, contact angle measurements, information on the permeability of the nanofibers compared to other materials, the influence of breathing parameters (inhalation-to-exhalation ratios, breath rate, incubation times), the optimization of the enzyme hydrogel in knife-coated hydrogel films and drop-coated hydrogel spots, the stabilization of the enzyme over time and enzyme leakage from the hydrogel, as well as an interference study and a comparison of fiber-based lactate capture and analysis to glucose analysis (PDF)

## †

Fraunhofer IZI-BB, Institute for Cell Therapy and Immunology, Department of Bioanalytics and Bioprocesses, Am Muehlenberg 13, 14476 Potsdam, Germany (B.V.G. and N.W.)

## ABBREVIATIONS

BSA: bovine serum albumin
COPD: chronic obstructive pulmonary disease
CVs: coefficients of variations
DMSO: dimethyl sulfoxide
EBC: exhaled breath condensate
GOx: glucose oxidase
HRP: horseradish peroxidase
HSA: human serum albumin
LOD: limit of detection
LOx: lactate oxidase
NFs: nanofibers
PAH: poly­(allylamine hydrochloride)
PB: polybrene
POC: point-of-care
SEM: scanning electron microscopy
TMB: 3,3′,5,5′-tetramethylbenzidin
WHO: world health organization
