# Supplementary material for: Engineered Nanofiber-Hydrogel Systems for Colorimetric Lactate Sensing from Breath
Source: ACS Appl Mater Interfaces. 2025 Nov 6;17(46):63009–19. doi: 10.1021/acsami.5c15741 (PMC12635974; doi:10.1021/acsami.5c15741)
Supplement: Supplementary file 1 [file am5c15741_si_001.pdf]

# SUPPORTING INFORMATION

# Engineered Nanofiber-Hydrogel Systems for Colorimetric Lactate Sensing from Breath

Autors:

*Barbara Grotz[a‡], Klara Rogalla von Bieberstein[a], Nongnoot Wongkaew[a], Axel Duerkop[a], Margaret W. Frey[b], Antje J. Baeumner[a]**

[a]*Institute of Analytical Chemistry, Chemo- and Biosensors, University of Regensburg, Universitaetsstrasse 31, 93053 Regensburg, Germany*

[b]*Department of Human Centered Design, College of Human Ecology, Cornell University, Ithaca, NY 14853, USA*

* Corresponding author: antje.baeumner@ur.de

**Summary of Supporting Information:**

| | |
|---|---|
| Number of pages | 24 |
| Number of tables | 4 |
| Number of figures | 21 |

**Table S 1:** Structures of the polymers used to produce positively charged nylon-PAH and nylon-PB.

| **Base-polymer** | **Cationic polymers/ Additives** |
|---|---|
| 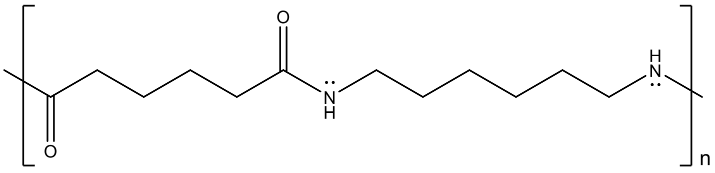 polyamide 6,6 (nylon) | 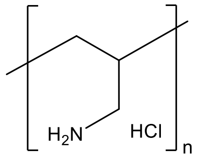 poly (allylamine hydrochloride) (PAH) 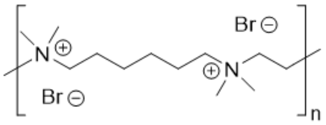 polybrene (PB) |

**Production the sensor discs with PET support**

Self-made adhesive support discs were produced with PET foils and double-sided adhesive tape. To assemble the discs for the free-standing design, the adhesive side of the support disc was placed on the nanofiber mat on a filter paper support, sandwiching the nanofibers between the adhesive and the filter paper. Free-standing nanofibers were obtained by carefully detaching the filter paper from the fibers. This step is facilitated by adding a drop of DI water on the filter paper.

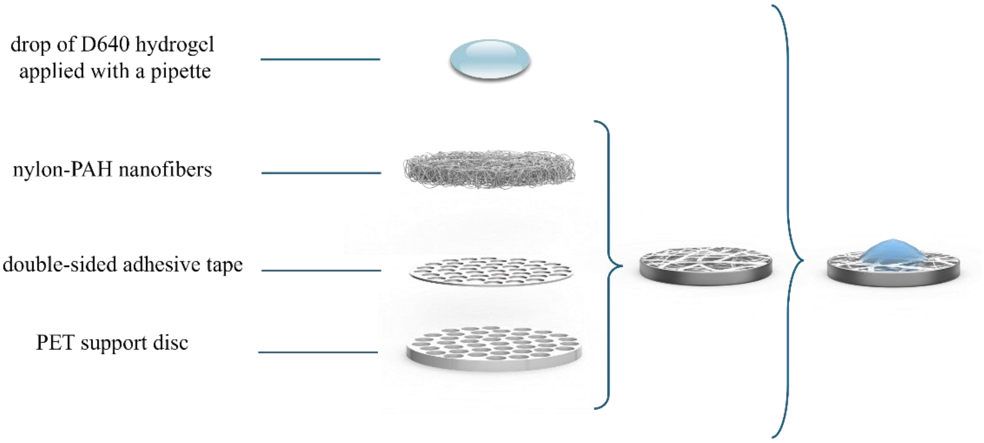


**Figure S 1:** Schematic drawing of the sample disc, consisting of the support disc for the free-standing nanofibers (PET support and double-sided adhesive tape), the nanofiber layer, and the hydrogel drop applied via drop coating containing the enzymes. Two sample types are

illustrated: nanofibers only, and nanofibers with hydrogel for combined sampling and detection (see Figure 5 in the main manuscript for assay overview).

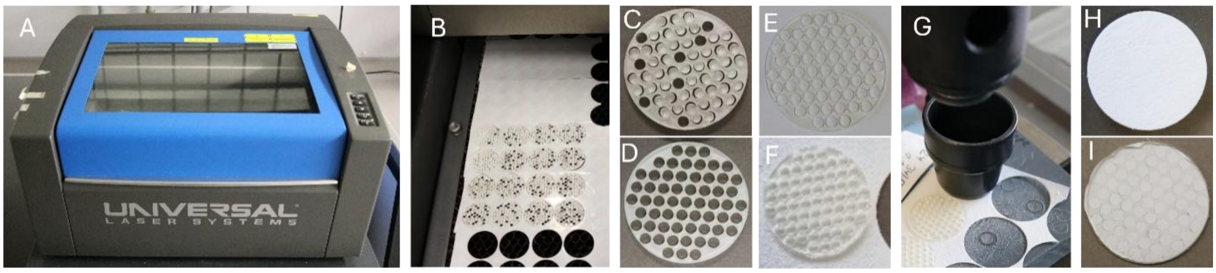


**Figure S 2:** Sample preparation procedure including the preparation of the support disc for the free-standing nanofiber sensing discs using a laser cutter (A-D) and the procedure of attaching the support to the nanofiber mat (E, F) as well as the cutting of the samples of the desired size out of the nanofiber mat (G) and the removal of the filter paper layer used as collection material in the electrospinning process (H) resulting in sample discs with freestanding nanofibers (I).

Vector graphics in CorelDraw suite 24.0 were used to design suitable support discs. Holes were implemented to allow stabilization of the nanofiber material in the breathing pathway, while ensuring/maintaining sufficient breathability. A colour code was used with red for cutting the material. The colour profile was set to RGB. The lines for laser cutting were drawn as hairlines without defined thickness, which is crucial for the vector mode. Double sided adhesive tape (SKS 50 mm x 50 m x 0,202 mm) was applied to PET foils (Modulor PET-G plates 0,5 x 200 x 400 mm) and the so prepared foils were placed in the laser cutter and cut following the prepared designs. A VLS 2.0 laser engraving system based on a 10600 nm infrared laser with a maximum power output of 30 W was used.

**Table S 2**: Laser settings for laser cutting.

| colour | function | speed / % | power / % | colour in RGB |
|---|---|---|---|---|
| red | cutting | 28 | 90-100 | 255:0:0 |

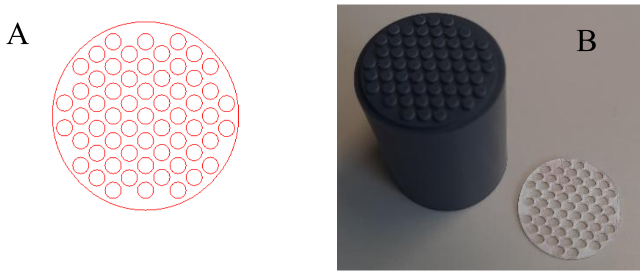


**Figure S 3:** Schematic drawing of the support disc for the free-standing nanofiber sensing discs used for laser cutting (diameter 23 mm) (A) and the picture of a support disc with the respective stamp (developed in house) to free the holes from remaining PET parts after laser cutting (B).

**Detailed Description of the Lactate Assay**

Captured lactate from aerosolized solutions was quantified using the well-known enzymatic reaction of LOx and HRP, with absorbance measurements of oxidized TMB. To optimize the assay in solution, different enzyme-TMB addition sequences were tested: (i) simultaneous (LOx+HRP+TMB), (ii) LOx pre-incubation before simultaneous addition of HRP and TMB (LOx/HRP+TMB), (iii) LOx+HRP pre-incubation before TMB addition (LOx+HRP/TMB), and (iv) sequential addition with individual 5-minute incubations (LOx/HRP/TMB) (Figure S 4). LOx+HRP/TMB yielded the best results, likely due to enzyme stabilization in solution and immediate hydrogen peroxide production, which rapidly oxidized TMB, producing sharp signals within 5 minutes. Among various solvents (DMSO, acetic acid, acetonitrile, THF) and buffer systems (citrate pH 6.0, DI water, MOPS pH 6.5, HEPES pH 7.4, PBS pH 8.0, TRIS pH 8.5) tested for TMB dissolution and dilution, DMSO and DI water proved most suitable (Figure S 16). In these solution-based assays, a defined enzyme solution (3.5 U LOx, 0.04 U HRP) was applied to nanofiber patches (diameter: 23 mm), followed by TMB addition (4 $\mu mol \cdot L^{-1}$) and incubation. Absorbance was measured at 655 nm, with signal enhancement at 450 nm after adding 50 µL of 0.5 $mol \cdot L^{-1}$ sulfuric acid, which improved the detection limit twofold (Figure 6) and served as a standard stopping step of the enzymatic reaction. Although the yellow product offers higher sensitivity, the blue coloration may eventually be better suited for point-

of-care detection, as untrained users can more easily distinguish different shades of blue (Figure S 5).

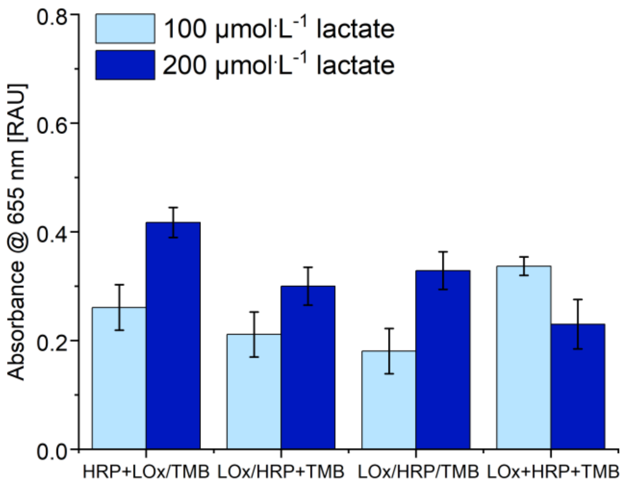


**Figure S 4**: Comparison of different assay set-ups of assay with enzyme solutions: HRP + LOx with subsequent TMB addition, LOx and subsequent HRP + TMB addition, separate, subsequent addition of LOx, HRP and TMB, combined addition of LOx, HRP and TMB.

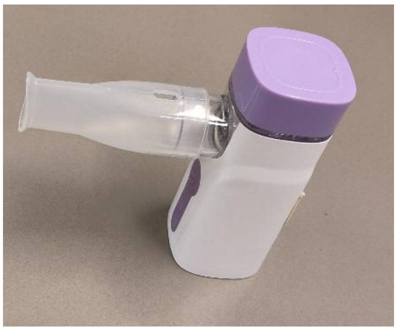

**Figure S 5**: Hand-held nebulizing device used in most experiments. It comprises of a chamber for the analyte solution (below purple cap) a membrane nebulizing the solution and a mouthpiece in which the sensing discs were introduced during incubation.

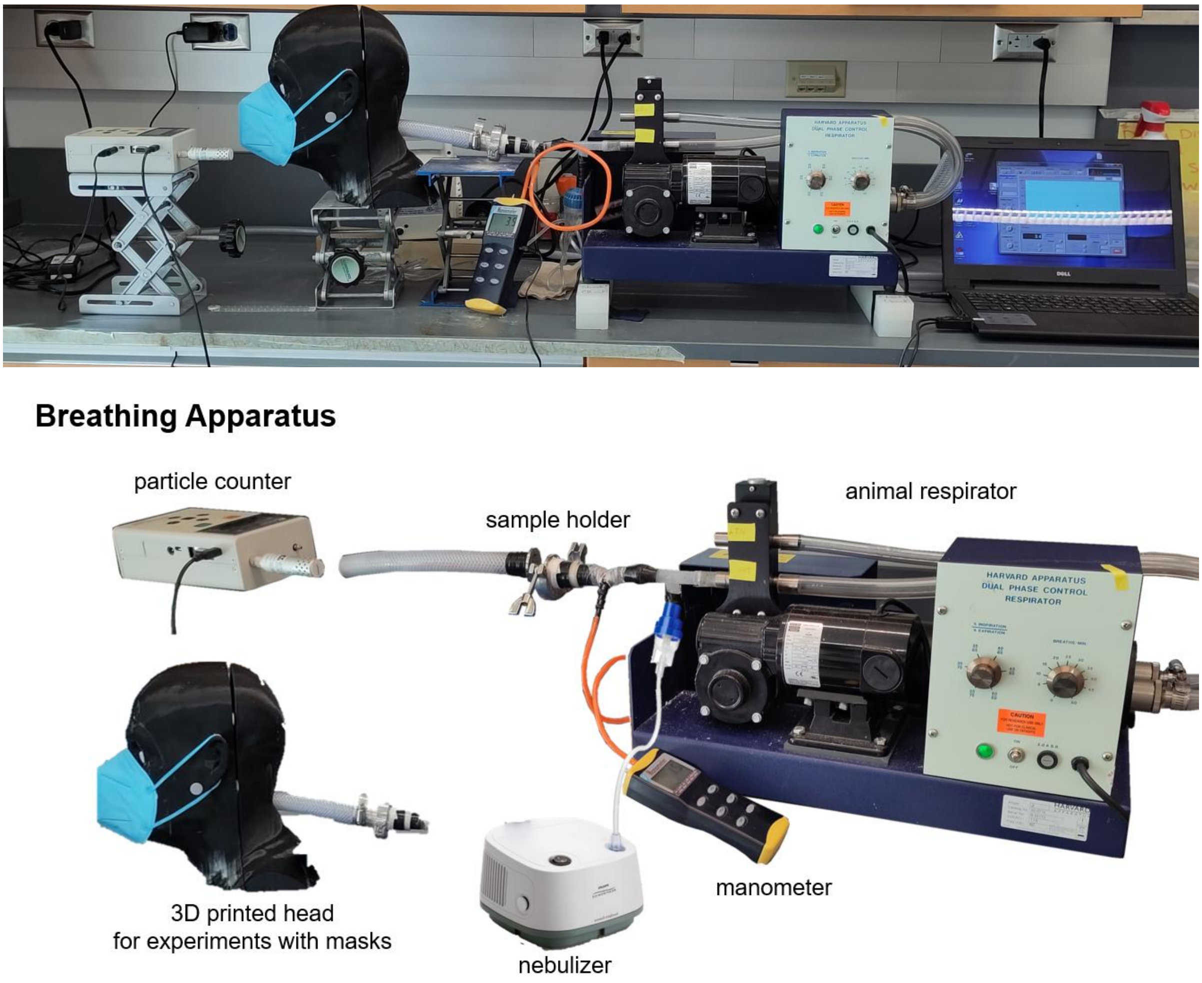


**Figure S 6**: Breathing apparatus used in breathing simulation experiments. It comprises of an animal respirator to adjust breath rate and inhalation/exhalation ratio and a sample holder which is interchangeable by a 3D-printed head where a face mask can be used. A manometer is included to measure the backpressure of the sample introduced via pressure measurements. A particle counter can also be introduced to the system, counting droplets of a size of 0.5 µm and 0.3 µm at the end of the sample holder/ other side of the mask. For analyte introduction a compressor with sample chamber is introduced in the breathing pathway of the respirator.

A

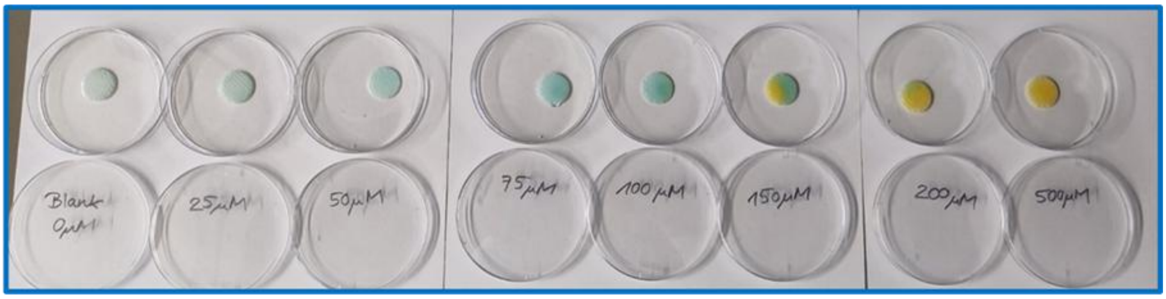


B

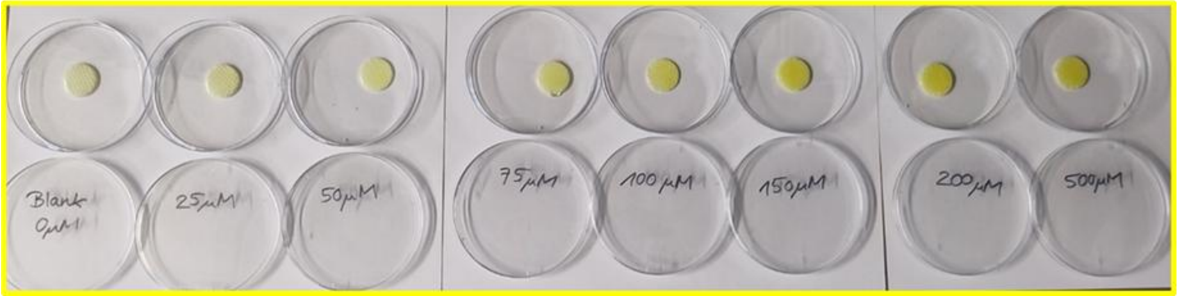


**Figure S 7**: Pictures of nanofiber patches after incubation with lactate vapour of different concentration and addition of LOx, HRP and TMB solutions A) before and B) after the stop of the reaction with sulfuric acid.

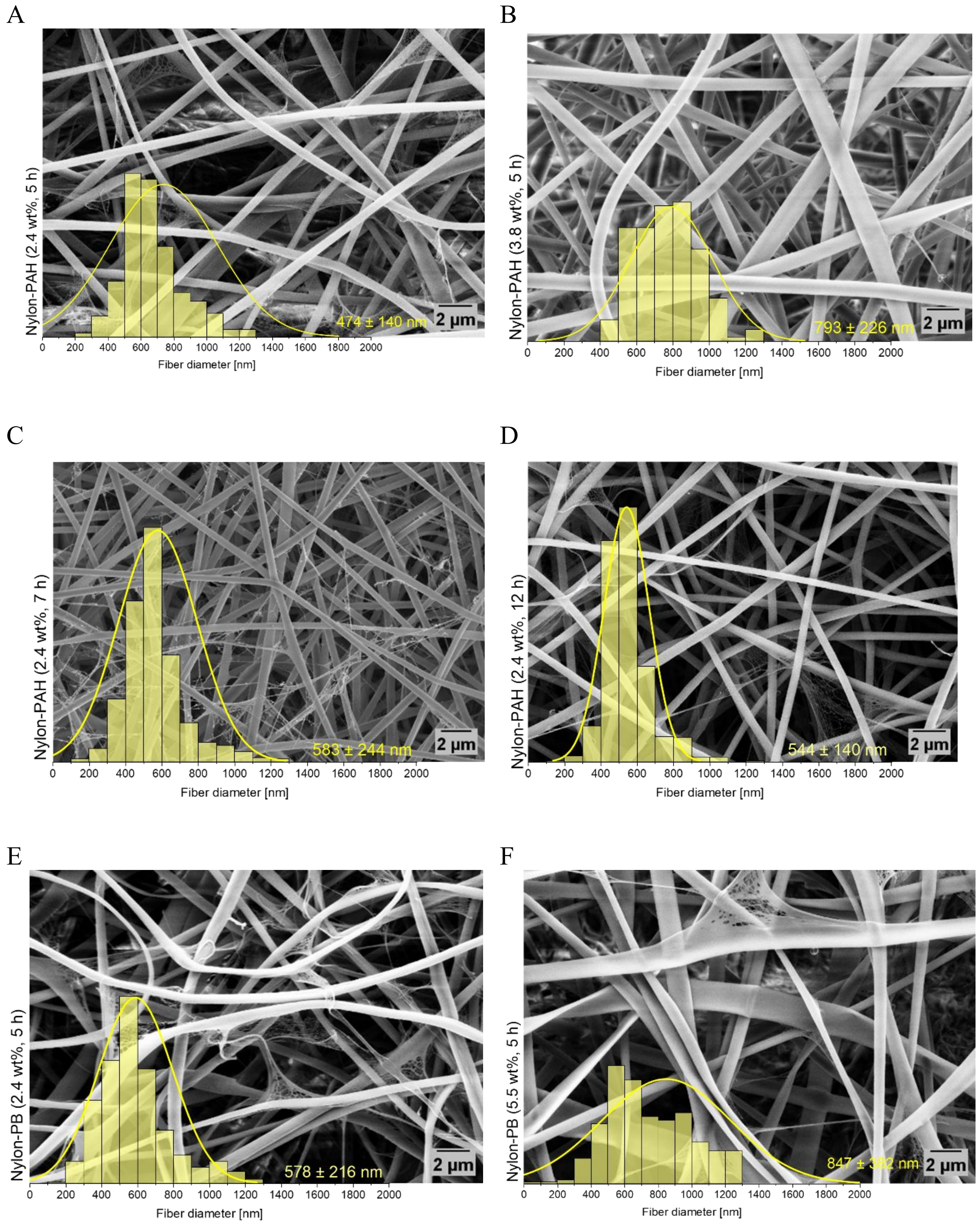


**Figure S 8**: Size distribution, mean fiber diameter and SEM images for a magnification of 10.00 kX for various nanofibers obtained by electrospinning, investigated as lactate capture elements for A-D) nylon-PAH and E-F) nylon-PB nanofibers differentiating in the choice of additive content (2.4 wt%, 3.8 wt% and 5.5 wt%) and spinning time (5 h, 7 h and 12 h).

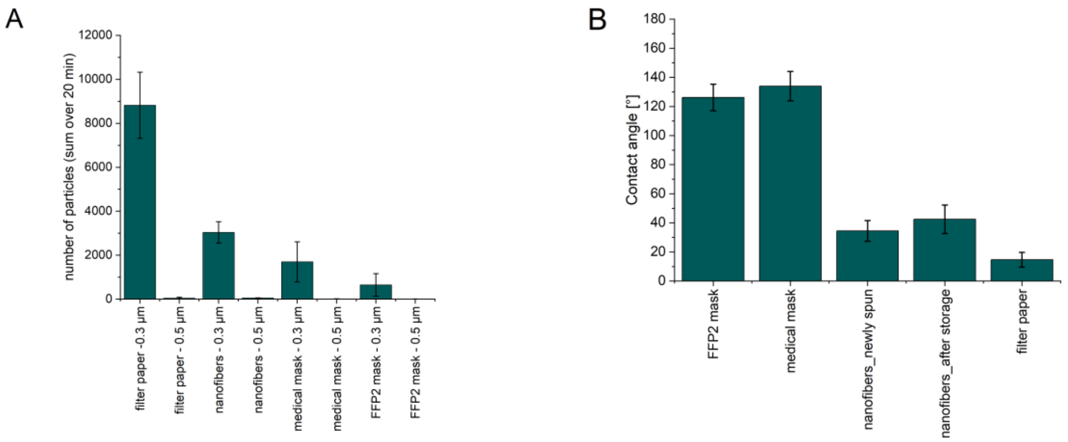


**Figure S 9:** Comparison of different materials (nylon-PAH nanofibers, filter paper, medical masks, FFP2/NS95 masks) with respect to A) filter efficiency (number of 0.3 µm and 0.5 µm particles leaking through the sample) and B) hydrophilic properties (given as contact angles).

Comparing the filter efficiencies of the materials to FFP2 masks focusing on particles of 0.3 µm diameter, filter paper filters 14 times less, nanofibers 5 times less and medical masks 3 times less particles (**Figure S 9 A**). The contact angle measurements showed hydrophobic behaviour for the mask materials with contact angles of 120-145°, whereas the hydrophilic properties of nylon-PAH-nanofibers were confirmed showing contact angles of 35° ± 15°. The storage of the nanofiber mats for 6 months did not significantly alter the hydrophilicity. Filter paper was used as positive control, showing immediate and complete wetting and a contact angle of 0° (**Figure S 9 B**).

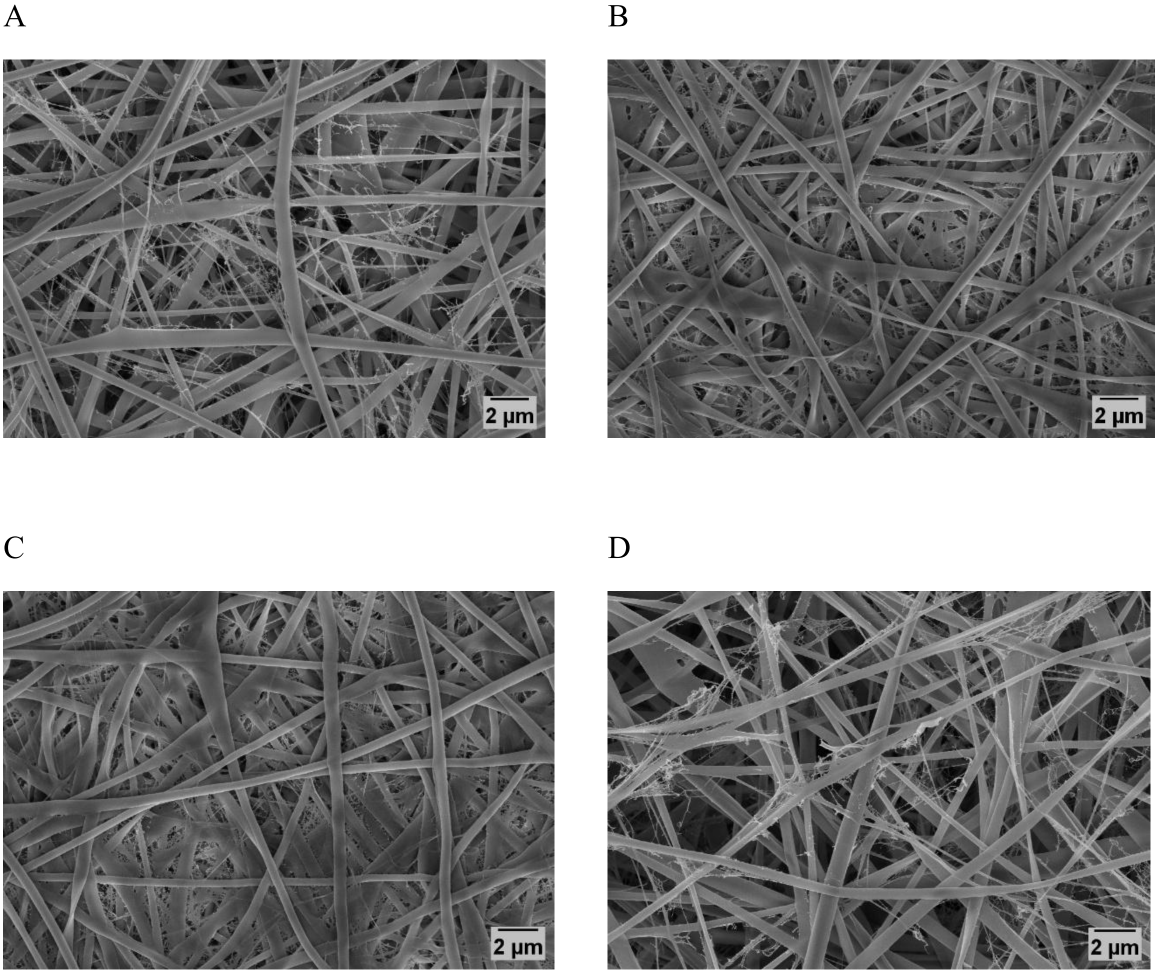


**Figure S 10:** SEM picture of nylon-PAH-nanofibers A) before and B, C) after incubation with lactate aerosol in the nebulizer. Mechanical influence during breathing on fiber morphology is negligible. Wetting of the nanofibers results in denser structure and slightly compressed fibers, while nanonets stay intact. D) SEM of nylon-PAH-nanofibers after storage for 8 months. The influence of storage on nanofiber morphology is negligible, fiber diameter and nanonet structure stay intact even after prolonged storage periods.

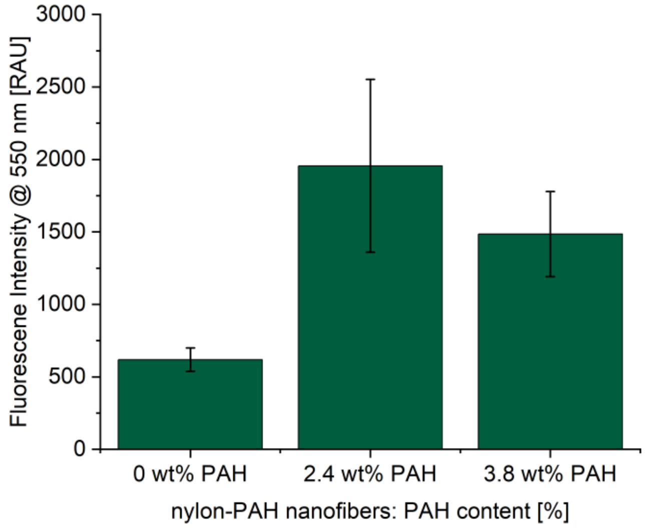


**Figure S 11:** CBQCA assay determining the $NH_2$-residues on nylon and nylon-PAH nanofibers with different PAH content (2.4 wt%, 3.8 wt%). n=4. The standard deviations are due to scattering effects resulting from the presence of the nanofibers in fluorescence measurements. Due to the binding chemistry of the CBQCA assay the signal producing moiety is covalently coupled to the nanofibers, and hence measurements of the fluorophore in solution (without the NF mat) are not possible.

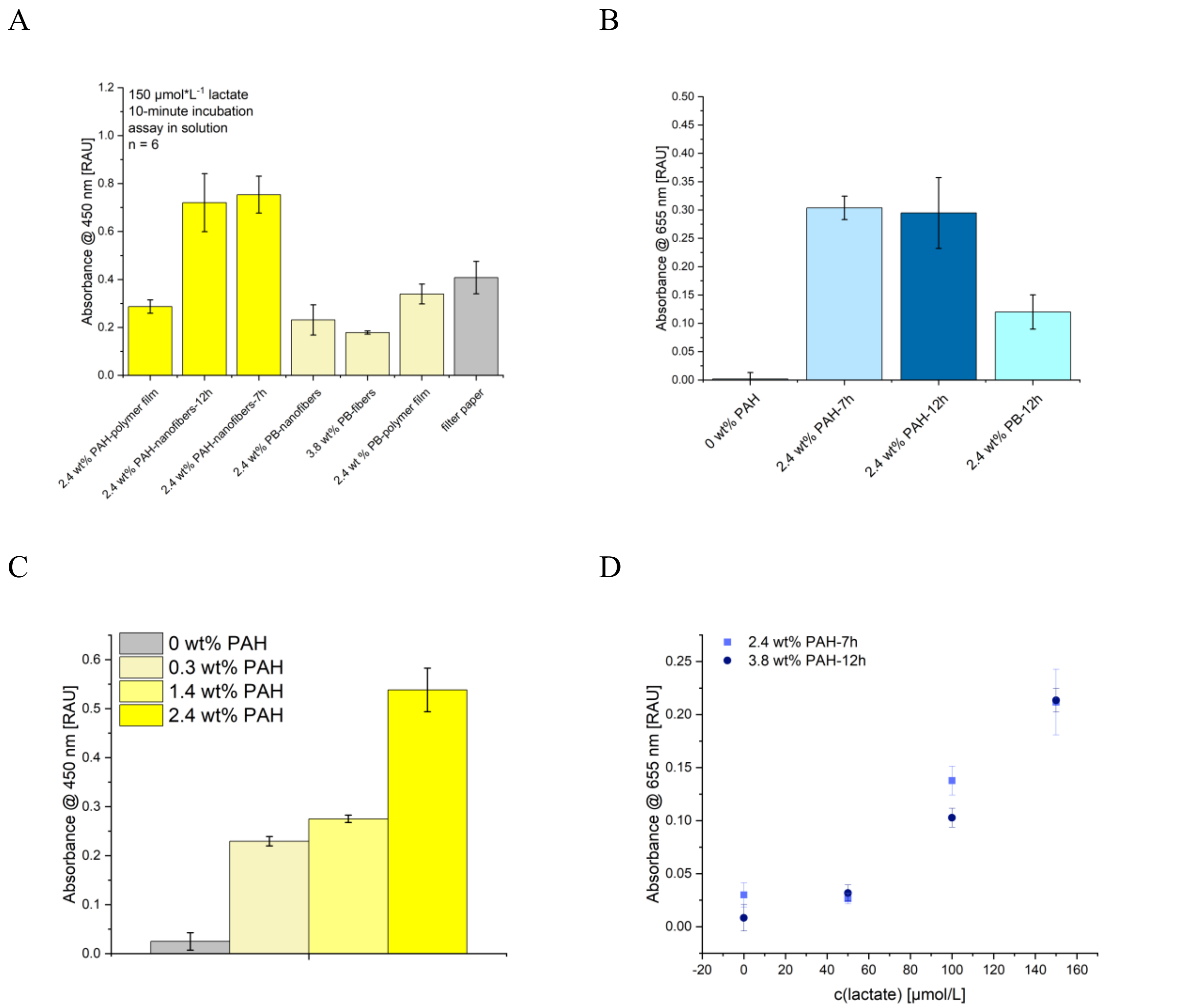


**Figure S 12:** Comparison of A) polymer films and nanofibers for lactate capture in MTP lactate assays and B) different electrospinning times (7 h, 12 h) for nanaofibers containing different additives (PAH or PB) and C) Optimization of PAH content (0.3 wt%, 1.4 wt% and 2.4 wt%) and D) spinning times (7 h and 12 h) for nylon-PAH nanofibers. Incubation in nebulizer for 2 minutes with 150 µmol·L$^{-1}$ (B-D). n=4

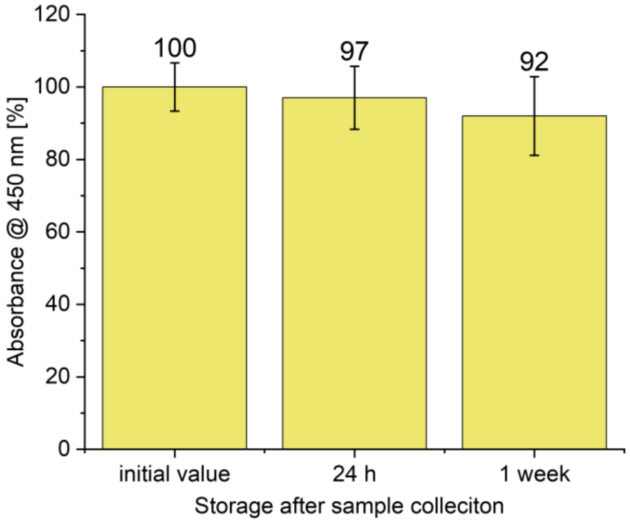


**Figure S 13:** Absorbance recovery for different storage times (24 h and 1 week) of nylon-PAH nanofibers after incubation with lactate aerosol. c(lactate):150 µmol·$L^{-1}$, LOx, HRP and TMB were added in solution prior to measurement. n=3.

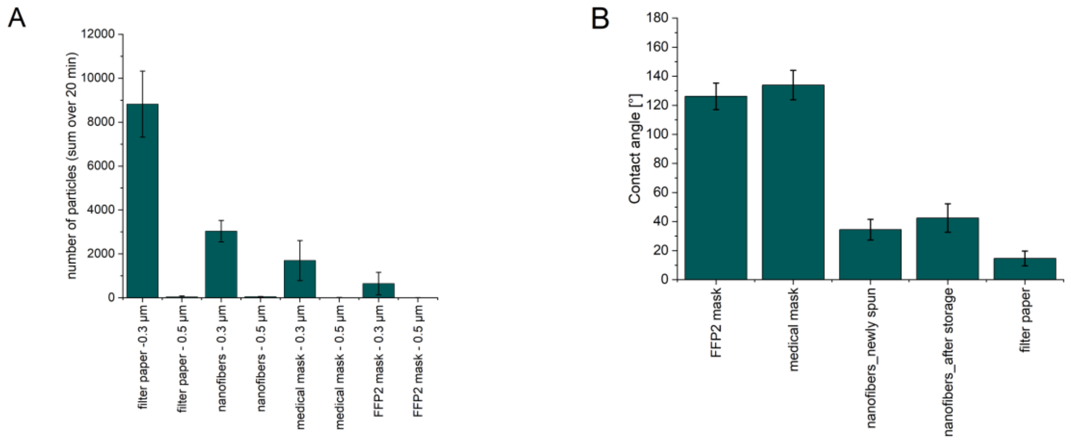


**Figure S 14:** Comparison of different materials (nylon-PAH nanofibers, filter paper, medical masks, FFP2/NS95 masks) with respect to A) filter efficiency (number of 0.3 µm and 0.5 µm particles leaking through the sample) and B) hydrophilic properties (given as contact angles).

Comparing the filter efficiencies of the materials to FFP2 masks focusing on particles of 0.3 µm diameter, filter paper filters 14 times less, nanofibers 5 times less and medical masks 3 times less particles (**Figure S 13 A**). The contact angle measurements showed hydrophobic behaviour for the mask materials with contact angles of 120-145°, whereas the hydrophilic

properties of nylon-PAH-nanofibers were confirmed showing contact angles of 35° ± 15°. The storage of the nanofiber mats for 6 months did not significantly alter the hydrophilicity. Filter paper was used as positive control, showing immediate and complete wetting and a contact angle of 0° (**Figure S 13 B**).

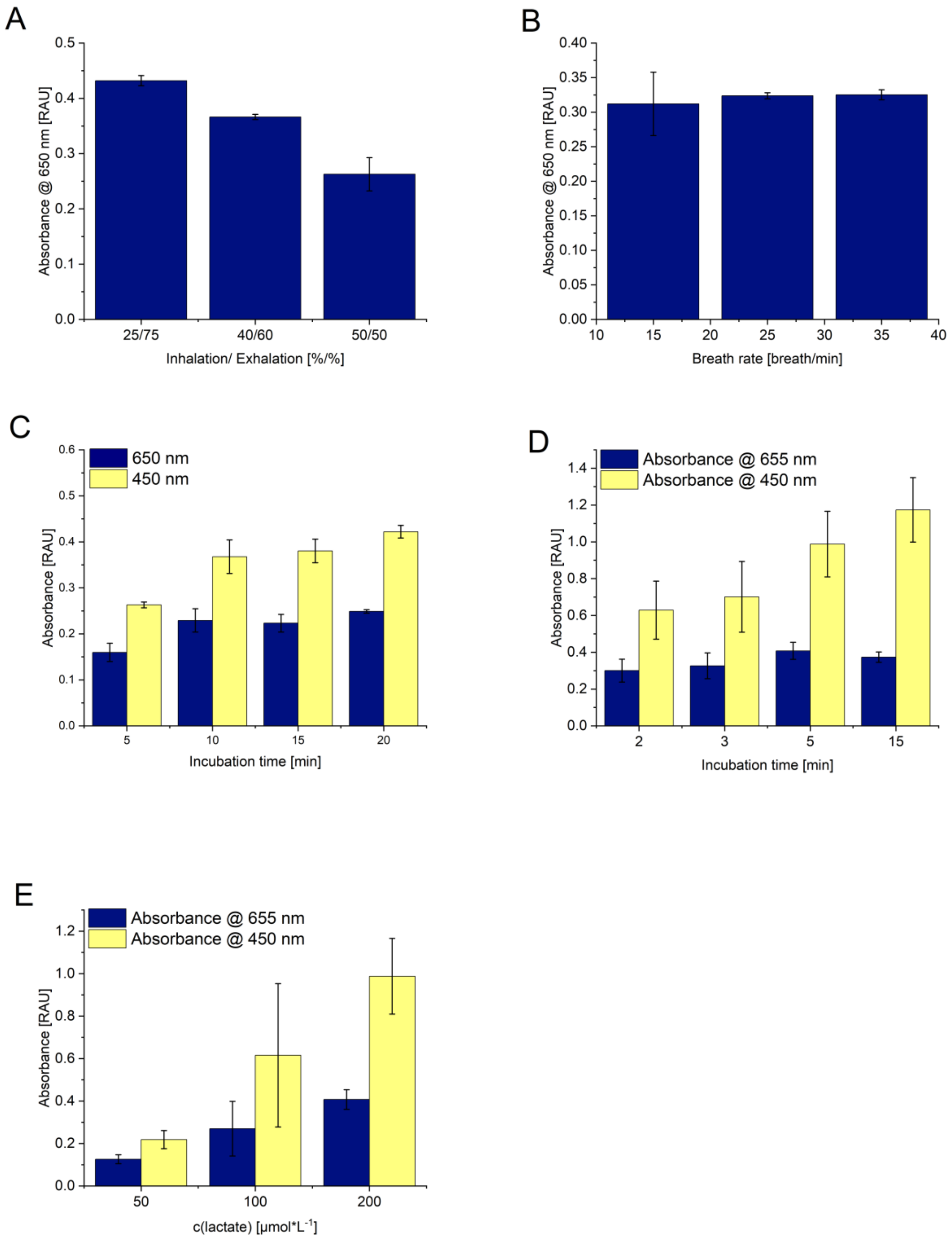


**Figure S 15**: Influence of A) varying inhalation to exhalation ratios (25/75, 40/60, 50/50), B) breath rates (15, 25 and 35 breaths/minute), C) varying sampling times in the simulated breathing apparatus (breath rate of 25 breaths/min, inhalation/exhalation ratio of 50/50; 5, 10, 15 and 20 minutes of sampling), D) varying sampling times in the hand-held nebulizing device (2, 3, 5 and 15 minutes) on the lactate capture efficiency of nylon-PAH nanofibers and E)

different analyte concentrations on the signal intensity for a sampling period of 5 minutes. A-C: 100 $\mu mol \cdot L^{-1}$of lactate, n=4. D: 200 $\mu mol \cdot L^{-1}$of lactate, n=3 from nanofiber mats produced over the course of a year.

The more analyte vapor is transferred onto the samples, longer sampling times in C and D or higher exhalation ratio in A (**Figure S 12**), the more intensive is the coloration. Indicating that increased sampling times can further increase signal intensities in the future allows fine tuning of the system comparing convenience (shorter sampling) and higher signal intensities (lower LODs). A sampling duration of 5 minutes already allowed the differentiation between 50 $\mu mol \cdot L^{-1}$, 100 $\mu mol \cdot L^{-1}$ and 200 $\mu mol \cdot L^{-1}$ lactate (**Figure S 12 E**). An increased number but shorter breaths per minute do not significantly influence the signal intensity. This is beneficial, as asthmatic patients usually breath with higher frequencies (**Figure S 12 B**) [1,2].

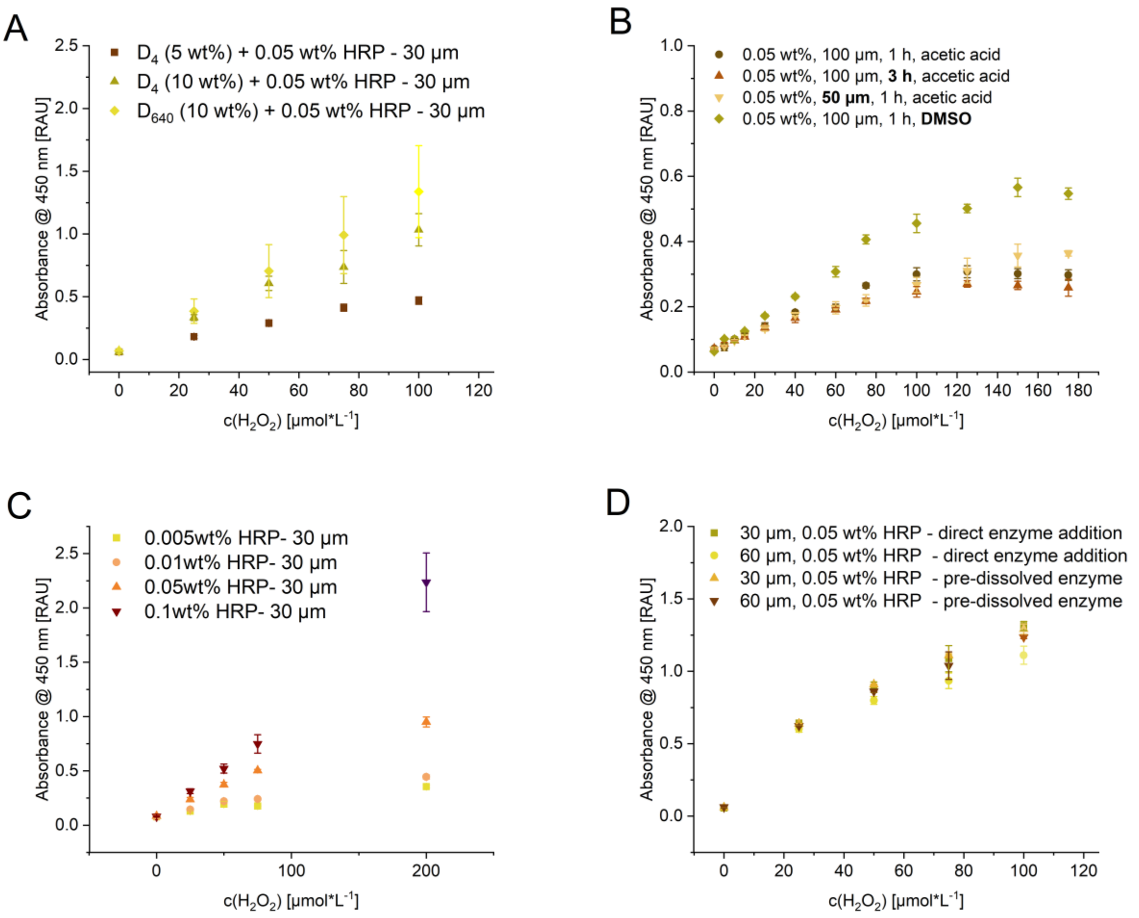


**Figure S 16**: Optimization of enzyme-hydrogel with respect to A) different hydrogel matrices (D4 (5wt%), D4 (10wt%), D640 (10wt%)) B) drying times (1 h, 3 h), solvent used for TMB stock solution (acetic acid, DMSO) and foil thickness (30 µm, 50 µm, 60 µm, 100 µm C) HRP concentration (0.005wt%, 0.01wt%, 0.05wt%, 0.1wt%) D) the different techniques in obtaining the enzyme-polymer mixture (addition of pre-dissolved enzyme or solid enzyme to the polymer solution). n=4.

Dissolution of TMB in DMSO resulted in higher signals compared to acetic acid. Higher enzyme concentrations led to lower LODs, while foil thickness did not significantly impact sensitivity. Improved sensitivities were observed with D640 compared to D4. Variations in hydrogel concentrations (10 wt% vs. 5 wt%) did not affect signal outcomes. While pre-dissolving enzymes reduced stirring times for homogeneous polymer-enzyme mixtures, it did not influence signal intensities. n=4.

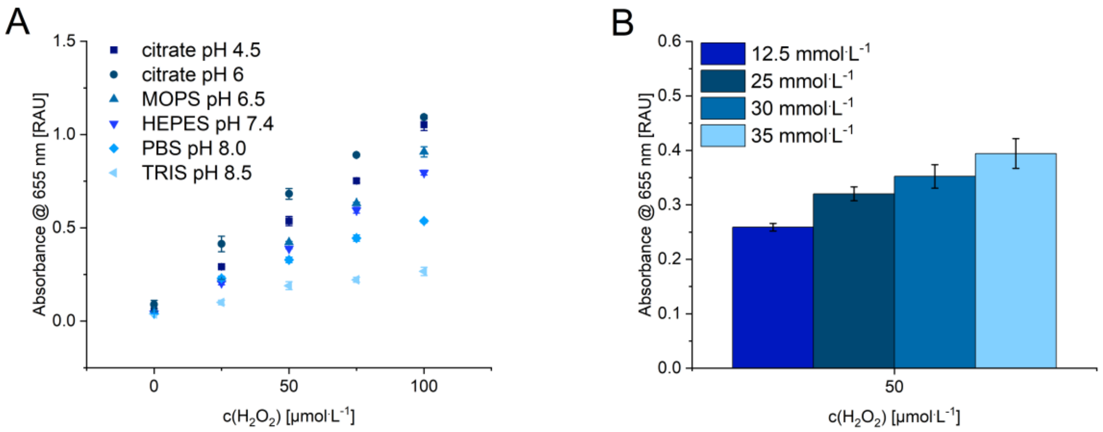


**Figure S 17:** Optimization of the HRP/TMB assay A) using different buffers (citrate, MOPS, HEPES, TRIS, PBS) for the dilution of TMB and B) using different TMB concentrations. n=4.

Lower pH values of the buffer solutions were identified as beneficial for dissolving TMB stock solutions (**Figure S 14 A**). Doubling the TMB concentration increased the signal by only 20%, so the 12.5 mmol·L$^{-1}$ TMB stock was used in further experiments to minimize costs (**Figure S 14 B**).

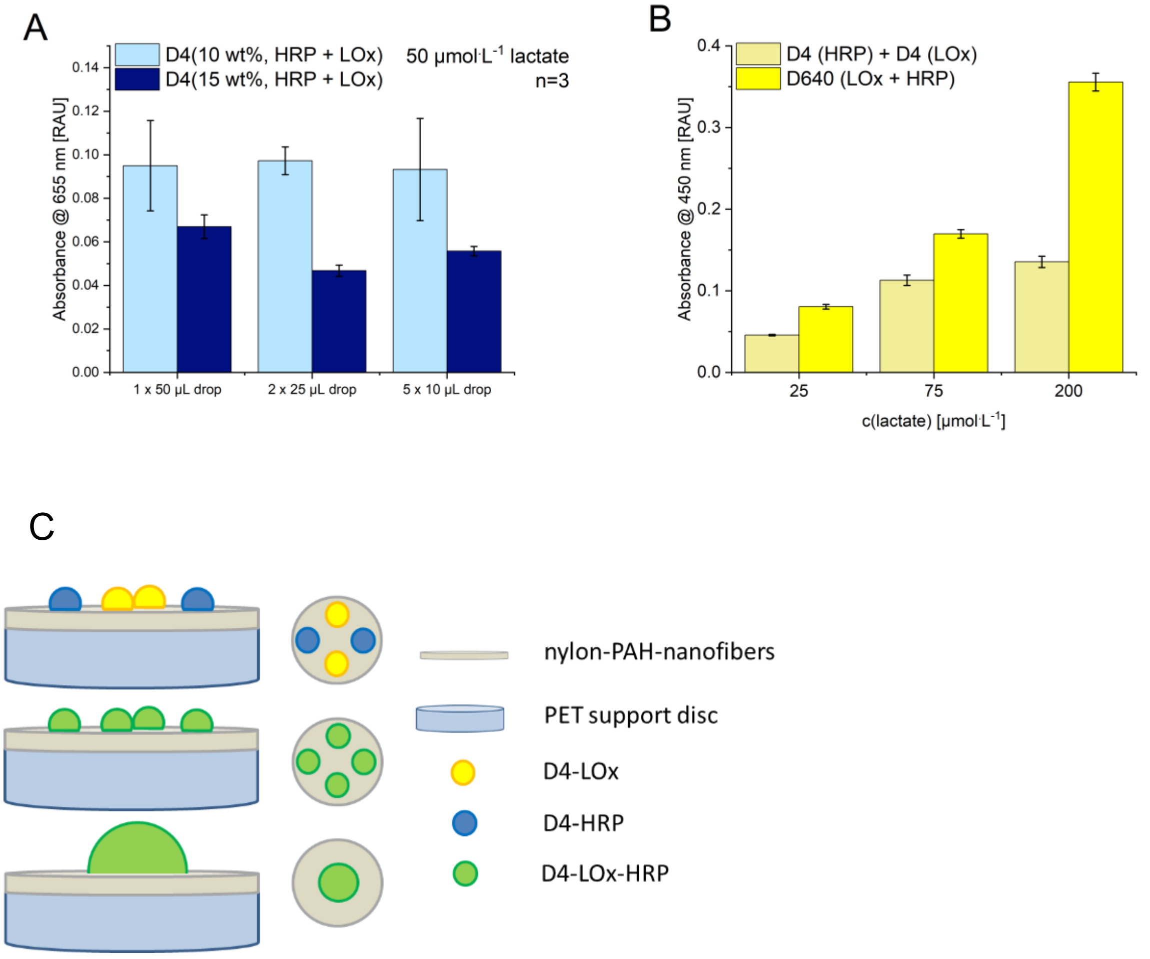


**Figure S 18:** A: Comparison of different drop sizes (10, 25 and 50 µL) and drop numbers (single drop vs. two drops) of D4-hydrogel- enzyme hydrogel (10 vs. 15 wt%) on nylon-PAH nanofibers. B: Combination of nylon-PAH nanofibers with enzyme hydrogels with both enzymes in one drop of D640-hydrogel (dark) and a set-up with separate drops of D4-hydrogel containing either LOx or HRP (light). C: Schematic scheme (side and top view) of the different drop-coating set-ups tested using D4 as exemplary hydrogel.

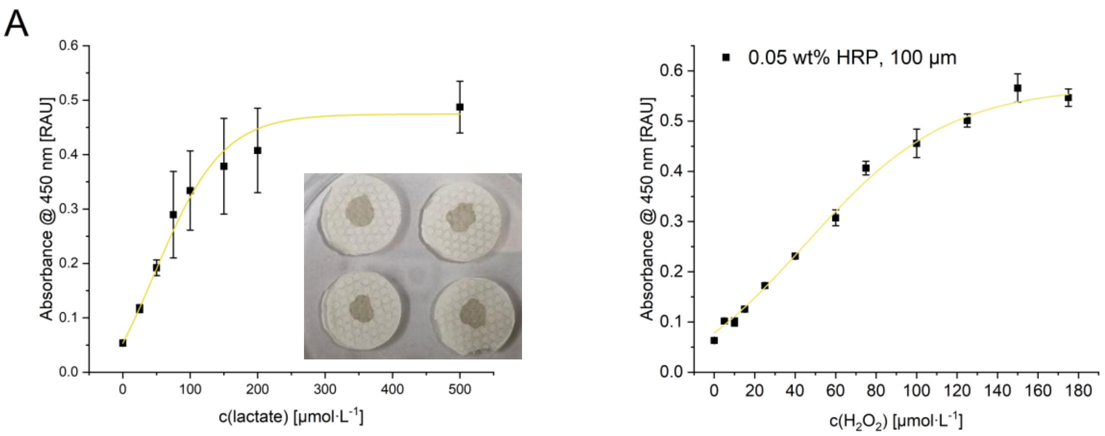


**Figure S 19**: A: Investigation of drop-coating reproducibility. n=16. Pictures of the individual drops on different nanofiber patches are shown. B: Dose response curve of HRP-D4-hydrogel under optimized assay conditions. n=4.

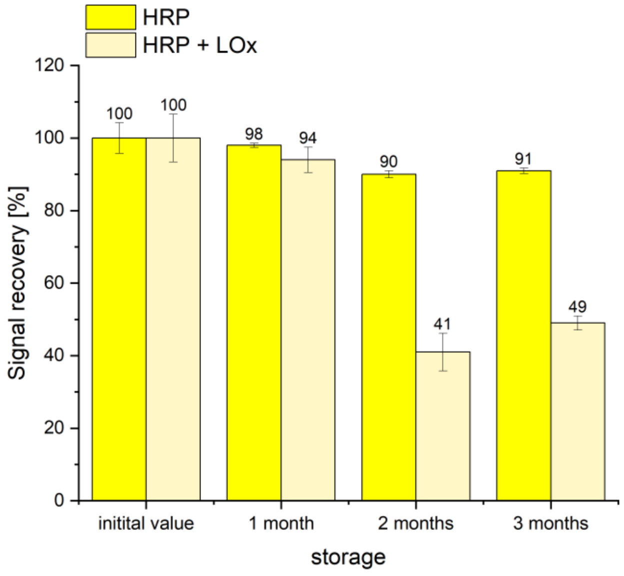


**Figure S 20:** Enzyme stability in D640-hydrogel for HRP and the combined immobilization of HRP and LOx over the course of 3 months plotted via the signal recovery in %. Storage at 4°C, c(lactate): 150 µmol·$L^{-1}$, n=4.

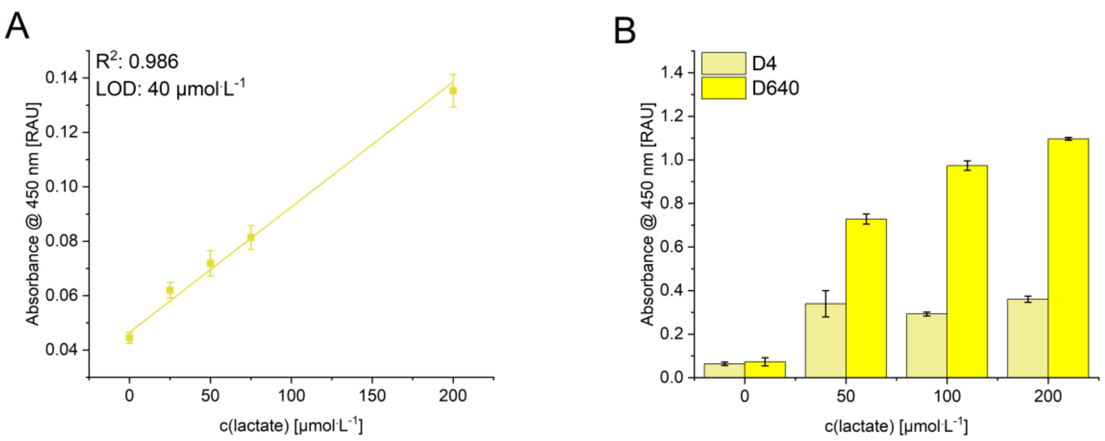


**Figure S 21:** A: Stability and functionality of HRP in D640 (10wt%, EtOH/$H_2O$) hydrogel (A, B) and D4 (10wt%, EtOH/$H_2O$) (B) measured via absorbance signals @450 nm for 4-months-old LOx-HRP-hydrogels in LOx+HRP/TMB assays with 0, (25,) 50, 75/100 and 200 µmol·L$^{-1}$ lactate after further LOx addition (20 µU LOx). Storage at 4°C, n=4.

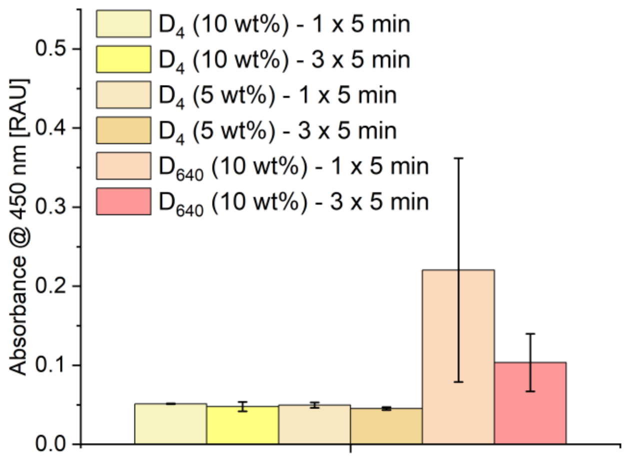


**Figure S 22**: Leakage study for D640-hydrogel (10 wt%) and D4-hydrogel (5, 10 wt%) with an enzyme content of 0.05 wt% Absorbance values at 450 nm were measured for collected washing solutions after washing of the hydrogels for 5 minutes or 3 times 5 minutes with fresh washing solutions (200 µL DI water) to determine the enzyme activity in the washing solutions and hence the leakage of enzymes from the hydrogels. Enzyme-hydrogel foils of a wet thickness

of 50 µm were used. n=4 of 6 mm samples per well were tested in a MTP. The signal obtained for the 5-minute long washing step for D640 showed high standard deviations and seemed unreliable in context of the other results.

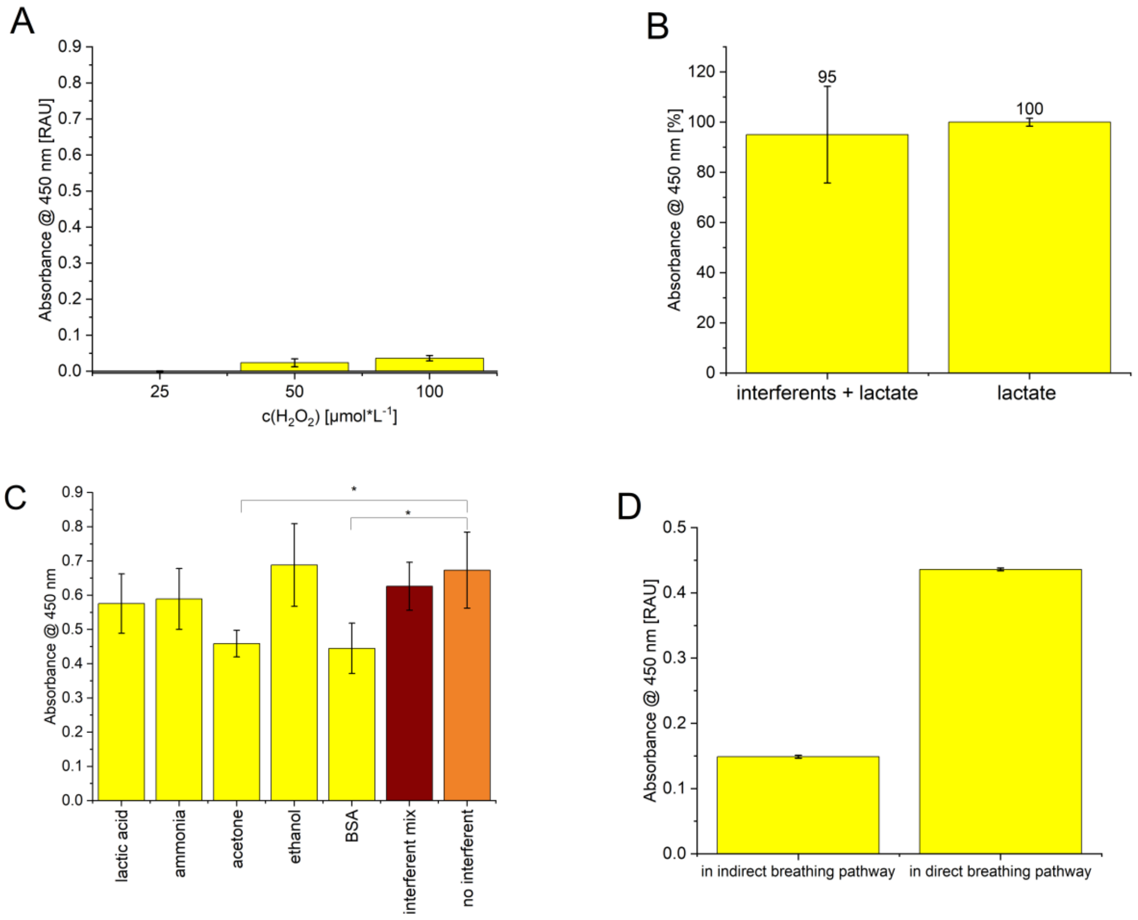


**Figure S 23:** Interference study with A) different $H_2O_2$ solutions, B) a mixture of different interferents present in breath, simulating breath aerosol and C) a second experiment comparing the influence of the interferents mixed vs. their influence when added to the sensing discs separately. The signals are corrected for background signals. In D) the difference between the position of the sample in the breathing pathway (in the indirect breathing pathway, equals on the side of a facemask vs. in the direct breathing pathway in front of the mouth or in the mouthpiece of the nebulizer) was investigated.

Signals obtained for different hydrogen peroxide (HP) concentrations (25, 50, and 100 $\mu mol \cdot L^{-1}$) were negligibly small (absorbance intensities <0.05 AU, **Figure S 20 A**),

indicating that HP levels in breath (0–10 μmol·L$^{-1}$) do not affect the lactate assay. Additionally, the presence of a mixture of potential interferents in breath (**Table S 3, Figure S 20 B**), including lactic acid, glucose, HP, HSA, ethanol, and acetone, did not influence signal intensities (95±10% signal recovery in presence of interferents). Individual addition of potential interferents such as BSA or acetone at elevated concentrations significantly affected lactate signals (**Figure S 20 C**), likely due to surface interactions with the sensing disc. However, the interference mixture produced signals comparable to controls, indicating negligible impact within the relevant concentration range found in exhaled breath. In contrast, the positioning of the sensing patch within the breathing pathway had a significant impact on signal intensity, highlighting the need for careful placement in future assay development (**Figure S 20 D**).

**Table S 3:** Overview of used interferents and their concentration used (bold) in the interference mix (**Figure S 20**) vs. their concentration range present in breath [3,4]. A lactate concentration of 150 μmol·L$^{-1}$was used in the interference mix containing lactate.

| **$H_2O_2$** | **ethanol** | **ammonia** | **acetone** |
|---|---|---|---|
| 0-<br>10 μmol·L$^{-1}$ | 0.2–0.6 nmol·L$^{-1}$<br>after<br>consumption:<br>200-500 nmol·L$^{-1}$ | 4-18 nmol·L$^{-1}$ | 1-25 ppm/ |
| **10 μmol·L$^{-1}$** | **10 μmol·L$^{-1}$** | **100 μmol·L$^{-1}$** | **1000 ppm** |
| **lactic acid** | **glucose** | **buffer<br>pH 6.8** | **BSA/ protein** |
| 150 nmol·L$^{-1}$-1 mmol·L$^{-1}$ | 0.2 μmol·L$^{-1}$-<br>2 mmol·L$^{-1}$ | 5-20 μmol·L$^{-1}$ | 1 μg·mL EB<br>C$^{-1}$ |
| **1.1 mmol·L$^{-1}$** | **2 mmol·L$^{-1}$** | **100 μmol·L$^{-1}$** | **1 μg·mL$^{-1}$** |

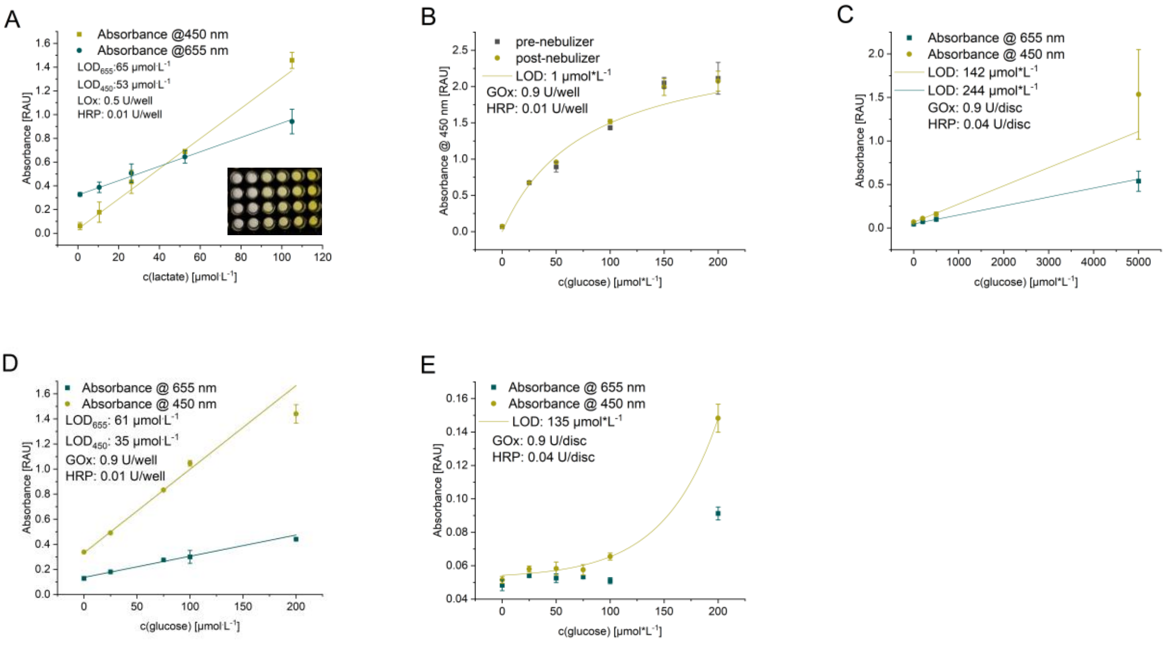


**Figure S 24**: Comparison of different assay set-ups featuring A, B) enzymes and analyte in solution C) nanofibers with GOx-solution D) analyte solution with GOx-hydrogel and E) nanofibers with GOx-hydrogel.

Comparative experiments with glucose further confirmed that both analyte capture efficiency and analyte-enzyme interaction are key determinants of sensitivity. While glucose detection relies solely on enzyme–analyte interactions, the lactate system benefits from electrostatic interactions that efficiently capture and accumulate analyte from breath aerosol over time, thereby up-concentrating the target and significantly enhancing system sensitivity (10 to 30-fold increase compared to glucose **Figure S 24, Figure 6**). If reproducible hydrogel-drop formation can be ensured, such as in industrial production settings, a further increase in sensitivity in hydrogel-based systems is expected.

**Table S 4**: Costs per assay for the two approaches tested with low and increased LOx concentrations. The values were calculated based on research-laboratory size containers of the material (polymers, hydrogel, solvents, enzymes, reagents). Prices were obtained on the Sigma Aldrich website. In case for also calculating the labour/ working hours and the minimal wage paid in Germany (12.84) was used. Prices are given in €.

| **costs per assay** | **with TMB and hydrogel** | |
|---|---|---|
| **LOx-low** | 2.75 | 0.29 |
| **LOx-high** | 3.43 | 0.97 |
| | with labour | without labour |

References


(1) Tobin, M. J.; Chadha, T. S.; Jenouri, G.; Birch, S. J.; Gazeroglu, H. B.; Sackner, M. A. Breathing patterns. 2. Diseased subjects. *Chest* **1983**, *84* (3), 286–294. DOI: 10.1378/chest.84.3.286.

(2) van Oosten, M.; Johnsen, A.; Magnusson, B.; Gudjonsdottir, M. Assessing ventilatory efficiency at rest in asthma: A longitudinal comparison with healthy subjects. *Physiological reports* **2025**, *13* (15), e70490. DOI: 10.14814/phy2.70490.

(3) Vasilescu, A.; Hrinczenko, B.; Swain, G. M.; Peteu, S. F. Exhaled breath biomarker sensing. *Biosensors & bioelectronics* **2021**, *182*, 113193. DOI: 10.1016/j.bios.2021.113193. Published Online: Mar. 26, 2021.

(4) Karyakina, E. E.; Lukhnovich, A. V.; Yashina, E. I.; Statkus, M. A.; Tsisin, G. I.; Karyakin, A. A. Electrochemical Biosensor Powered by Pre-concentration: Improved Sensitivity and Selectivity towards Lactate. *Electroanalysis* **2016**, *28* (10), 2389–2393. DOI: 10.1002/elan.201600232.
